# *Tm*DorX2 positively regulates antimicrobial peptides in *Tenebrio molitor* gut, fat body, and hemocytes in response to bacterial and fungal infection

**DOI:** 10.1038/s41598-019-53497-4

**Published:** 2019-11-14

**Authors:** Maryam Keshavarz, Yong Hun Jo, Ki Beom Park, Hye Jin Ko, Tariku Tesfaye Edosa, Yong Seok Lee, Yeon Soo Han

**Affiliations:** 1 Department of Applied Biology, Institute of Environmentally-Friendly Agriculture (IEFA), College of Agriculture and Life Sciences, Chonnam National University, Gwangju, 61186 Republic of Korea; 2 Department of Life Science and Biotechnology, College of Natural Sciences, Soonchunhyang University, Asan, South Korea

**Keywords:** Antimicrobial responses, Innate immunity, RNAi

## Abstract

Dorsal, a member of the nuclear factor-kappa B (NF-κB) family of transcription factors, is a critical downstream component of the Toll pathway that regulates the expression of antimicrobial peptides (AMPs) against pathogen invasion. In this study, the full-length ORF of *Dorsal* was identified from the RNA-seq database of the mealworm beetle *Tenebrio molitor* (*TmDorX2*). The ORF of *TmDorX2* was 1,482 bp in length, encoding a polypeptide of 493 amino acid residues. *Tm*DorX2 contains a conserved Rel homology domain (RHD) and an immunoglobulin-like, plexins, and transcription factors (IPT) domain. *TmDorX2* mRNA was detected in all developmental stages, with the highest levels observed in 3-day-old adults. *TmDorX2* transcripts were highly expressed in the adult Malpighian tubules (MT) and the larval fat body and MT tissues. After challenging the larvae with *Staphylococcus aureus* and *Escherichia coli*, the *TmDorX2* mRNA levels were upregulated 6 and 9 h post infection in the whole body, fat body, and hemocytes. Upon *Candida albicans* challenge, the *TmDorX2* mRNA expression were found highest at 9 h post-infection in the fat body. In addition, *TmDorX2*-knockdown larvae exposed to *E. coli*, *S. aureus*, or *C. albicans* challenge showed a significantly increased mortality rate. Furthermore, the expression of 11 AMP genes was downregulated in the gut and fat body of ds*TmDorX2*-injected larvae upon *E. coli* challenge. After *C*. *albicans* and *S. aureus* challenge of ds*TmDorX2*-injected larvae, the expression of 11 and 10 AMPs was downregulated in the gut and fat body, respectively. Intriguingly, the expression of antifungal transcripts *TmTenecin-3* and *TmThaumatin-like protein-1* and *-2* was greatly decreased in *TmDorX2*-silenced larvae in response to *C. albicans* challenge, suggesting that *TmDorX2* regulates antifungal AMPs in the gut in response to *C. albicans* infection. The AMP expression profiles in the fat body, hemocytes, gut, and MTs suggest that *TmDorX2* might have an important role in promoting the survival of *T. molitor* larvae against all mentioned pathogens.

## Introduction

Insects are confronted by a variety of complex and evolving pathogens, and require a sophisticated and strong innate immune system because they lack adaptive immunity^1^. Besides primary defenses, that include integument and gut epithelium barriers, the insect innate immune system comprises cellular and humoral defense responses^2^. Humoral immunity, which includes Toll and immune deficiency (Imd) signaling pathways, regulates the synthesis of potent antimicrobial peptides (AMPs)^3^.

The Toll and Imd signaling pathways are triggered once immune-related tissues (e.g., the fat body) sense pathogen-associated molecular patterns (PAMPs) through pathogen recognition receptors (PRRs)^4,5^. The interaction of PAMPs with PRRs activates the intracellular Toll and Imd signal cascades leading to nuclear translocation of nuclear factor kappa-light-chain-enhancer of activated immune cells (NF-κB) transcription factors such as dorsal and dorsal-related immunity factor (Dif) and Relish for the Toll and Imd pathways, respectively. Ultimately, NF-κB in association with other factors regulate the transcription of AMP genes^6,7^.

Proteins in the NF-κB family share a strikingly well-conserved structure called the Rel homology domain (RHD), which is known to be involved in DNA binding, dimerization, nuclear localization, and interaction with the inhibitor κB (IκB)^8^. Based on the presence of multiple copies of ankyrin repeats (ANK) or transactivation domains (TAD) at the C-terminus of RHD, NF-κB proteins are classified into Class I and II, respectively.

Dorsal, a class II NF-κB protein, has been identified in different insects such as beetles^9^, flies^10^, mosquitoes^11,12^, bees^13^, and silk worms^14^. *Dorsal* (14 kb) was initially identified as maternal transcript essential for the embryonic establishment of dorsal-ventral polarization^10^, and was later described to engage in the immune response against infection by Gram-positive bacteria and fungi^15^. In *Aedes aegypti* larvae and female mosquitoes, levels of the *Dorsal* homolog, *Aa*REL1, are markedly increased after *Enterobacter cloacae*, *Micrococcus luteus*, and *Beauveria bassiana* challenge^11^. *Dorsal* silencing experiments have shown that the Toll pathway regulates immune responses against Gram-positive (*Paenibacillus larvae*) and Gram-negative bacteria (*E. coli*) infection in honey bee (*Apis mellifera*) pupae^13^.

In addition to its function in insects, *Dorsal* expression and its role in innate immunity has been noted in crustacean species. In the Chinese shrimp (*Fenneropenaeus chinensis*), antibacterial responses to *Micrococcus lysodeikticus* (Gram-positive bacteria) and *Vibrio anguillarum* (Gram-negative bacteria) were mediated by *Fc*Dorsal^16^, whereas in the Pacific white shrimp (*Litopenaeus vannamei*), *Lv*Dorsal regulates the *penaeidin-*4 gene (antifungal and anti-Gram positive bacterial AMP)^17,18^. Experiments in the crab model *Eriocheir sinensis*, showed that *EsDorsal* was primarily expressed in hemocytes and engaged in antifungal and antibacterial immune responses^19^. Furthermore, in aquatic invertebrates such as the cyclopoid copepod, *Paracyclopina nana, Dorsal* and *Dorsal-like* genes show immune function against stressful environmental conditions^20^.

The mealworm beetle *Tenebrio molitor* (Coleoptera) is a reliable model to study host-pathogen interactions during bacterial^21–23^ and fungal^24^ infection. In the last decade or so, extracellular events in the *T. molitor* Toll signaling pathway have been addressed in detail^25,26^. Contrary to its mammalian counterparts, Toll requires the endogenous ligand spätzle (Spz) for AMP production in insects such as *T. molitor*, *Drosophila*, and *Manduca sexta*^26–28^. The functional form of Spz is formed when the activated Spz-processing enzyme (SPE) cleaves the pro-protein Spz (pro-Spz). At the last step of the protease cascade, Spzinteracts with the ectodomain of the transmembrane receptor^29,30^. A recent study has shown that *Toll-like receptor 7* from *T. molitor* (*TmToll-7*) has anti-*E. coli* activity inducing expression of antibacterial AMPs^31^. In fact, we have identified nine extracellular ligands, namely *TmSpz-1b*, *−3*, *−4*, *−*5, *−*6, *−*7, *−7a*, *−7b*, *7b* and *-like* (unpublished data). However, the ligand-binding partner for *TmToll-7* remains to be elucidated. Furthermore, biochemical studies have shown that lysine-type peptidoglycan (Lys-type PGN) is recognized by the *Tenebrio* peptidoglycan recognition protein (PGRP-SA/Gram-negative binding protein (GNBP-1) complex^32^. Moreover, Gram-negative binding protein 3 (*Tm*GNBP3) plays a pivotal role in the induction of *TmTenecin-1* after *Beauveria bassiana* infection^33^. Furthermore, the intracellular Toll adaptor protein *Tm*MyD88 has been implicated in the Toll signaling cascade^34^. Additionally, an early study from our team has characterized Cactin from *T. molitor* (*Tm*Cactin), a protein interacting with a Cactus homolog, and described its role as a positive regulator of the Toll pathway^35^. However, the events downstream of *Tm*MyD88 have not yet been elucidated yet in *T. molitor*. The main purpose of this study was to analyze the developmental and tissue-specific expression level of *TmDorX2* and to determine its mRNA expression patterns in response to bacterial and fungal insults. Moreover, we sought to clarify the effect of *TmDorX2* silencing on AMP gene expression and survivability of *T. molitor* larvae in response to *E. coli*, *S. aureus*, and *C. albicans* challenge.

## Materials and Methods

### *T. molitor* rearing and culture of microbial strains

The mealworm *T. molitor* was reared on an artificial diet consisting of 170 g wheat flour, 0.5 g chloramphenicol, 20 g roasted soy flour, 0.5 g sorbic acid, 0.5 ml propionic acid, 10 g soy protein, and 100 g wheat bran, in 200 ml of distilled water which was autoclaved at 121 °C for 20 min. The insects were kept in an insectary at 27 ± 1 °C, and 60 ± 5% relative humidity (RH) in the dark. Healthy larvae at the 10^th^–12^th^ instar (approximately 2.4 cm) were used for experiments. The Gram-negative bacteria *Escherichia coli* (strain K12), Gram-positive bacteria *Staphylococcus aureus* (strain RN4220), and the fungus *Candida albicans* were obtained from the American Type Culture Collection (ATCC) and used for immune challenge experiments. *C. albicans* suspension was prepared by culturing the fungi in Sabouraud Dextrose broth, whereas *E. coli* and *S. aureus* were cultivated in Luria-Bertani (LB) broth at 37 °C overnight. In order to use microorganisms for challenge experiments, overnight cultures were harvested, washed 3 times, and suspended in phosphate-buffered saline (PBS; pH 7.0). Subsequently, the cultures were centrifuged at 3,500 rpm for 15 min and OD_600_ values were measured using a spectrophotometer (Eppendorf, Germany). Based on the measured OD_600_ values, the microorganism cultures were adjusted to 10^6^ cells/μl for *E. coli* and *S. aureus*, and 5 × 10^5^ cells/μl for *C. albicans*.

### Bioinformatics analysis for *TmDorX2* identification and sequence characterization

To identify *TmDorX2*, a local-tblastn analysis was performed using the *Tribolium castaneum* Dorsal 2 (*Tc*Dorsal 2) amino acid sequence (GenBank: EFA02885.1) as the query, against the *T. molitor* nucleotide database. The deduced amino acid sequence was subjected to InterProScan 5.0^36^ and blastx^37^ analyses for specific domain analysis predictions. The nuclear localization signal (NLS) was predicted by cNLS Mapper^38^. The selected amino acid sequences of each insect species, which include 5 beetles, 4 bees, 3 ants, 3 flies,3 mosquitos, 2 moths, 1 butterfly, and 1 stink bug (Table 1), were aligned using ClustalX2^39^ to estimate the amino acid sequence similarity between *TmDorX2* and its orthologs. The MEGA 7.0 program was utilized to create a phylogenetic tree^40^ using the maximum-likelihood (ML) method and the JTT matrix-based model^41^. The Dorsal protein of the Chinese white shrimp *Penaeus chinensis* (*Pc*Dorsal: ACJ36225.1) was used as an outgroup in the phylogenetic studies. A bootstrap analysis with 1000 replicates was used to derive the confidence of branches in the phylogenetic tree.

**Table 1:** The accession number of Dorsal proteins used for bioinformatic analysis of this study.

Name	Abbreviation	GenBank Number
*Drosophila melanogaster* dorsal, isoform D	*Dm*DorD	NP_001163000.1
*Aedes albopictus* embryonic polarity protein dorsal-like	*Aa*Dor	XP_019931750.1
*Anopheles glabripennis* embryonic polarity protein dorsal-like isoform X3	*Ag*Dor	XP_018572644.1
*Apis florea*; embryonic polarity protein dorsal-like isoform X3	*Af*Dor	XP_012343274.1
*Apis mellifera* dorsal protein isoform X2	*Am*Dor	XP_006567065.1
*Apis dorsata* embryonic polarity protein dorsal-like isoform X5	*Ad*DorX5	XP_006619742.1
*Bombus terrestris* embryonic polarity protein dorsal isoform X2	*Bt*Dor	XP_012174122.1
*Nicrophorus vespilloides* putative transcription factor p65 homolog isoform X2	*Nv*Dor	XP_017781152.1
*Asbolus verrucosus* dorsal, partial	*Av*Dor	RZB54393.1
*Tribolium castaneum* Dorsal	*Tc*Dor	EFA02850.1
*Tribolium castaneum* Dorsal 2	*Tc*Dor2	EFA02885.1
*Trachymyrmex cornetzi* embryonic polarity protein dorsal isoform X3	*Tc*DorX3	XP_018357420.1
*Halyomorpha halys* embryonic polarity protein dorsal-like isoform X6	*Hh*DorX6	XP_014275495.1
*Pogonomyrmex barbatus* embryonic polarity protein dorsal isoform X3	*Pb*DorX3	XP_006567063.1
*Monomorium pharaonis* embryonic polarity protein dorsal isoform X2	*Mp*DorX2	XP_011647341.1
*Delia antiqua* Dorsal	*Da*Dor	AFI98401.1
*Anopheles darlingi* Rel1/Dif/Dorsal	*Ad*Dor	ETN66814.1
*Bombyx mori* embryonic polarity protein dorsal isoform A	*Bm*DorA	NP_001166296.1
*Spodoptera litura* embryonic polarity protein dorsal isoform X4	*Sl*DorX4	XP_022815079.1
*Pieris rapae* embryonic polarity protein dorsal isoform X3	*Pr*DorX3	XP_022116742.1
*Culex quinquefasciatus* embryonic polarity protein dorsal	*Cq*Dor	XP_001844078.1
*Anopheles sinensis* Dorsal isoform 1-B	*As*Dor1-B	KFB39849.1
*Penaeus chinensis* dorsal	*Pc*Dor	ACJ36225.1

### Gene expression analysis of *TmDorX2* during different developmental stages and in various tissues

To elucidate the developmental pattern of *TmDorX2* mRNA expression, samples were randomly collected from eggs (EG), young larvae (YL; 10^th^–12^th^ instar), late instar larvae (LL; 19^th^–20^th^ instar), pre-pupae (PP), 1 to 7-day-old pupae (P1-P7), and 1- to 5-day-old adults (n = 20 for each stage). To elucidate the tissue-specific pattern of *TmDorX2* mRNA expression, larval and adult tissues of *T. molitor* including the fat body, Malpighian tubules (MTs), gut, integument, hemocytes, ovary, and testis, were dissected. Subsequently, total RNA was extracted from the collected samples following the LogSpin RNA isolation method with minor modifications^42^. Briefly, the tissue samples were homogenized in guanidine thiocyanate based RNA lysis buffer (20 mM EDTA, 20 mM MES buffer, 3 M guanidine thiocyanate, 200 mM sodium chloride, 40 μM phenol red, 0.05% Tween-80, 0.5% glacial acetic acid (pH 5.5), and 1% isoamyl alcohol in 50 ml) using a bead-based homogenizer (Bertin Technologies, France). After incubation at room temperature (approximately 25 °C) for 1 min, the samples were centrifuged at 15,000 rpm for 5 min at 4 °C. Subsequently, 100 μl from the supernatants were diluted in 200 μl of RNA lysis buffer added to 300 μl of 99.9% ethanol. and were centrifuged at 15,000 rpm for 30 s at 4 °C, using silica spin columns (Bioneer, Korea, KA-0133-1). The aqueous phase was discarded, and the genomic DNA was digested using DNase (Promega, USA, M6101) for 15 min at 37 °C. Subsequently, the silica spin columns were washed with 450 μl of 3 M sodium acetate buffer by centrifugation at 15,000 rpm for 30 s at 4 °C. Next, 500 μl of 80% ethanol was added to the spin columns and the samples were centrifuged again. After drying the spin column for 1 min, total RNA was eluted in 30 µl of distilled water for cDNA synthesis and other downstream applications. 2 μg of total RNAs were used for cDNA synthesis by using the AccuPower® RT PreMix (Bioneer, Korea) kit with an oligo-(dT)^12–18^ primer according to the manufacturer’s instructions.

The synthesized cDNAs (1:20 dilution with DNase/RNase free water) were used as template for quantitative reverse-transcription PCR (qRT-PCR). The qRT-PCR analyses of cDNA samples were performed in AccuPower® 2X GreenStar qPCR Master Mix (Bioneer), containing the specifically designed primers, *TmDorX2*-qPCR-Fw and *TmDorX2*-qPCR-Rv (Table 2) (Fig. S1). Primers for *TmDorX2* and *T. molitor ribosomal protein* (*TmL27a*), which was used as an internal control were designed using Primer 3.0 plus (http://www.bioinformatics.nl/cgi-bin/primer3plus/primer3plus.cgi). The PCR amplification conditions were as follows: 95 °C for 5 min, followed by 40 cycles at 95 °C for 15 s and 60 °C for 30 s. Finally, *TmDorX2* gene expression was evaluated using the comparative C_T_ method (2^−ΔΔCT^ method)^43^.

**Table 2:** Sequences of the primers used in this study.

Name	Primer sequences
TmDorX2_qPCR_Fw	5′-ACACCCCCGAAATCACAAAC-3′
TmDorX2_qPCR_Rv	5′-TTTCAGAGCGCCAGGTTTTG-3′
TmDorX2_Temp_Fw	5′- AAACCTGGCGCTCTGAAAC-3′
TmDorX2_Temp_Rv	5′-CAGGTGAATCGGTTGGAGTT-3′
dsTmDorX2_Fw	5′-TAATACGACTCACTATAGGGT CTATCTAGCTGGCAGGGACG-3′
dsTmDorX2_Rv	5′-TAATACGACTCACTATAGGGT CAGGTGAATCGGTTGGAGTT-3′
dsEGFP_Fw	5′-TAATACGACTCACTATAGGGT ACGTAAACGGCCACAAGTTC-3′
dsEGFP_Rv	5′-TAATACGACTCACTATAGGGT TGCTCAGGTAGTGTTGTCG-3′
TmTenecin-1_Fw	5′-CAGCTGAAGAAATCGAACAAGG-3′
TmTenecin-1_Rv	5′-CAGACCCTCTTTCCGTTACAGT-3′
TmTenecin-2_Fw	5′-CAGCAAAACGGAGGATGGTC-3′
TmTenecin-2_Rv	5′-CGTTGAAATCGTGATCTTGTCC-3′
TmTenecin-3_Fw	5′-GATTTGCTTGATTCTGGTGGTC-3′
TmTenecin-3_Rv	5′-CTGATGGCCTCCTAAATGTCC-3′
TmTenecin-4_Fw	5′-GGACATTGAAGATCCAGGAAAG-3′
TmTenecin-4_Rv	5′-CGGTGTTCCTTATGTAGAGCTG-3′
TmDefensin-1_Fw	5′-AAATCGAACAAGGCCAACAC-3′
TmDefencin-1_Rv	5′-GCAAATGCAGACCCTCTTTC-3′
TmDefencin-2_Fw	5′-GGGATGCCTCATGAAGATGTAG-3′
TmDefencin-2_Rv	5′-CCAATGCAAACACATTCGTC-3′
TmColeoptericin-1_Fw	5′-GGACAGAATGGTGGATGGTC-3′
TmColeoptericin-1_Rv	5′-CTCCAACATTCCAGGTAGGC-3
TmColeoptericin-2_Fw	5′-GGACGGTTCTGATCTTCTTGAT-3′
TmColeoptericin-2_Rv	5′-CAGCTGTTTGTTTGTTCTCGTC-3′
TmAttacin-1a_Fw	5′-GAAACGAAATGGAAGGTGGA-3′
TmAttacin-1a_Rv	5′-TGCTTCGGCAGACAATACAG-3′
TmAttacin-1b_Fw	5′-GAGCTGTGAATGCAGGACAA-3′
TmAttacin-1b_Rv	5′-CCCTCTGATGAAACCTCCAA-3′
TmAttacin-2_Fw	5′-AACTGGGATATTCGCACGTC-3′
TmAttacin-2_Rv	5′-CCCTCCGAAATGTCTGTTGT-3
TmCecropin-2_Fw	5′-TACTAGCAGCGCCAAAACCT-3′
TmCecropin-2_Rv	5′-CTGGAACATTAGGCGGAGAA-3′
TmThaumatin-like protein-1_Fw	5′-CTCAAAGGACACGCAGGACT-3′
TmThaumatin-like protein-1_Rv	5′-ACTTTGAGCTTCTCGGGACA-3′
TmThaumatin-like protein-2_Fw	5′-CCGTCTGGCTAGGAGTTCTG-3′
TmThaumatin-like protein-2_Rv	5′-ACTCCTCCAGCTCCGTTACA-3′
TmL27a_qPCR_Fw	5′-TCATCCTGAAGGCAAAGCTCCAGT-3′
TmL27a_qPCR_Rv	5′-AGGTTGGTTAGGCAGGCACCTTTA-3′

^※^Underline indicates T7 promoter sequences.

### Analysis of *TmDorX2* mRNA expression after microbial challenge

In order to determine *TmDorX2* mRNA expression upon microbial challenge, *T. molitor* larvae (10^th^–12^th^ instar larvae) were experimentally challenged by injecting 1 µl of *E. coli* (1 × 10^6^ cells/µl), *S. aureus* (1 × 10^6^ cells/µl), and/or *C. albicans* (5 × 10^4^ cells/µl) into separate groups of larvae. Immune tissues, including the fat body, hemocytes, gut, and MTs, were collected from each of the microbe-infected group and from PBS-injected groups acting as a wounding control at 3, 6, 9, 12, and 24 h post-infection. Subsequently, total RNA extraction, cDNA synthesis, and qRT-PCR were carried out as described above.

### *TmDorX2* gene silencing

To prepare double-stranded RNA against *TmDorX2* (ds*TmDorX2*), we designed forward and reverse primers containing the T7 promoter sequence at their 5′ ends using the SnapDragon-Long dsRNA design software (https://www.flyrnai.org/cgi-bin/RNAi_find_primers.pl) (Table 2) (Fig. S1). The 480-bp PCR product was amplified in AccuPower® Pfu PCR PreMix with the *TmDorX2*_Temp_Fw and *TmDorX2*_Temp_Rv (Table 2) (Fig. S1) at 95 °C for 2 min, followed by 30 cycles of denaturation at 95 °C for 20 s, annealing at 56 °C for 30 s, and extension at 72 °C for 5 min. The procedure was followed using the same PCR conditions, which led to production of a 388-bp PCR product containing the T7 promoter sequence (Table 2). The synthesized ds*TmDorX2* was purified, using an AccuPrep® PCR Purification Kit (Bioneer, Korea), precipitated with 5 M ammonium acetate, and washed with 70% ethanol. Subsequently, the PCR product was used as a template to synthesize ds*TmDorX2 in vitro* using an EZ^TM^ T7 High Yield *in vitro* Transcription Kit (Enzynomics, Korea) as per the manufacturer’s instructions. Briefly, 1 μg of the final PCR product was mixed with 4 μl of 5X Transcription Buffer, 2 μl of 10X MgCl_2_, 2 μl of DTT (100 mM), 1 μl of RNase Inhibitor (40 U/μl), 1 μl of rATP (100 mM), 1 μl of rGTP (100 mM), 1 μl of rCTP (100 mM), 1 μl of rUTP (100 mM), and 1 μl of T7 RNA polymerase. Subsequently, the mixture was incubated at 37 °C for 3 h and 25 °C for 1 h. The synthesized ds*TmDorX2* was mixed with one volume of 5 M ammonium acetate, incubated on ice for 15 min, and washed three times using 70%, 80% and 99.9% ethanol, respectively. Finally, after drying, the pellet was resuspended in 30 μl distilled water (Sigma, USA, W4502-1L). A 546 bp PCR product of the *Enhanced Green Fluorescent Protein (EGFP)* gene (derived from the plasmid EGFP-C1) was used as a template to synthesize double-stranded EGFP (ds*EGFP*) acting as negative control.

Then, to address the importance of *TmDorX2* in *T. molitor* humoral immunity, 1 μl (1 μg) of ds*TmDorX2* was injected into *T. molitor* 10^th^–12^th^ instar larvae. Control insects were injected with ds*EGFP*. The efficacy of ds*TmDorX2* gene silencing was confirmed by qRT-PCR at 2 days post-injection. After confirmation of silencing, dsRNA-injected larvae (n = 10 per group) were infected with *E. coli* (1 × 10^6^ cells/larva), *S. aureus* (1 × 10^6^ cells/larva), or *C. albicans* (5 × 10^4^ cells/larva), and mortality was monitored every day for a duration of 10 days. The experiment was repeated three times with 10 larvae per group for each experiment.

### Analysis of *TmDorX2* silencing on AMP expression post-microbial challenge

To elucidate the function of the protein encoded by the *TmDorX*2 transcript in regulating AMP genes, the gene expression profile of 14AMPs including *TmTenecin*-1 (*TmTene1*), *TmTenecin*-2 (*TmTene2*), *TmTenecin*-3 (*TmTene3*), *TmTenecin*-4 (*TmTene4*), *TmAttacin-1a* (*TmAtt1a*), *TmAttacin-1b* (*TmAtt1b*), *TmAttacin-2* (*TmAtt2*), *TmDefensin*-1 (*TmDef1*), *TmDefensin*-2 (*TmDef2*), *TmColeoptericin-1* (*TmCole1*), *TmColeoptericin-2* (*TmCole2*), *TmCecropin-2* (*TmCec2*), *TmThaumatin-like protein-1* (*TmTLP1*), and *TmThaumatin-like protein-2* (*TmTLP2*) were determined in the *TmDorX2*-silenced larvae after microbial challenge. ds*EGFP* was used as the negative control, and PBS served as the wound control. Experimental samples were dissected 24 h post-injection and the following immune-sensing tissues were isolated: the fat body, hemocytes, gut, and MTs. The samples were processed for cDNA synthesis, and qRT-PCR analysis was conducted using AMP-specific primers (Table 2).

### Statistical analysis

All experiments were carried out in triplicate and data were subjected to one-way analysis of variance (ANOVA). In order to evaluate the difference between groups (*p* < 0.05), the Tukey’s multiple range test was performed. The results for the mortality assay were analyzed using the Kaplan-Meier plot (log-rank Chi-square test) in Excel (http://www.real-statistics.com/survival-analysis/kaplan-meier-procedure/real-statistics-kaplan-meier/).

## Results

### Gene organization, cDNA analysis, and phylogenetic tree

**Figure 1:**
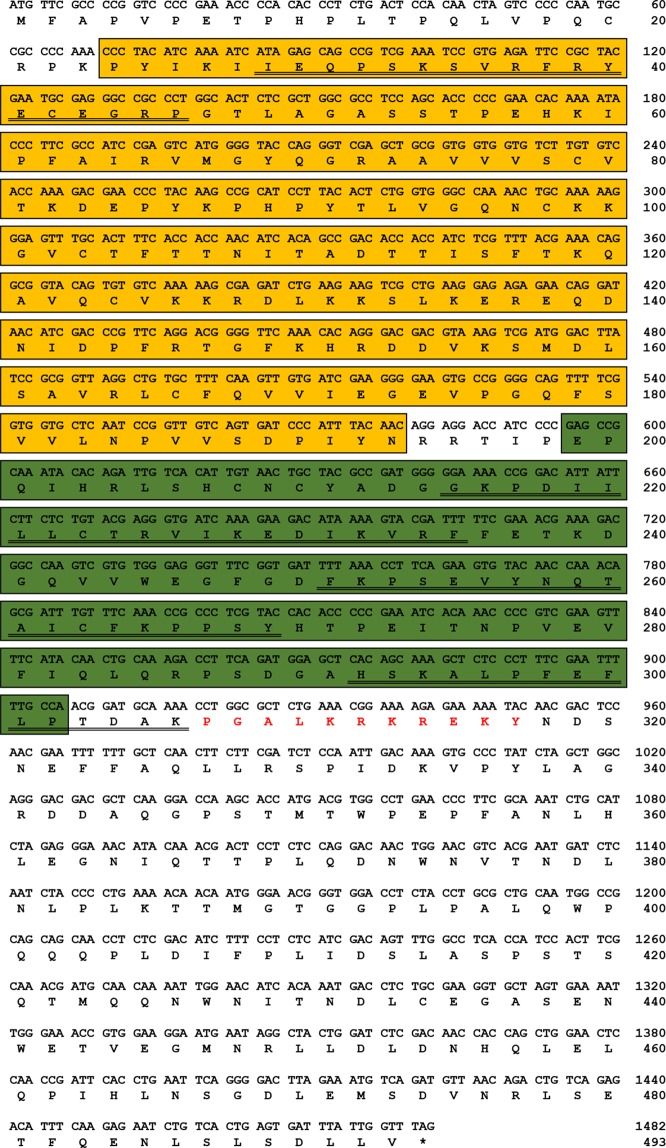
The full-length ORF sequence of the *T. molitor Dorsal* isoform X2 (*TmDorX2*) and its deduced amino acid sequence. Nucleotide and amino acid numbers are shown at the right margin, showing that the *TmDorX2* ORF sequence contains 1,482 bp nucleotides encoding 493 amino acid residues. The sequence at the N-terminal region is representing the Rel homology domain (RHD) is highlighted in the yellow box, and that of the immunoglobulin-like, plexins, transcription factors (IPT) domain is highlighted in the green box. The nuclear localization signal (NLS; P_307_GALKRKREKY_317_) is shown in red. The transcription factor NF-κB signature sequences are indicated with a double underline.

**Figure 2:**
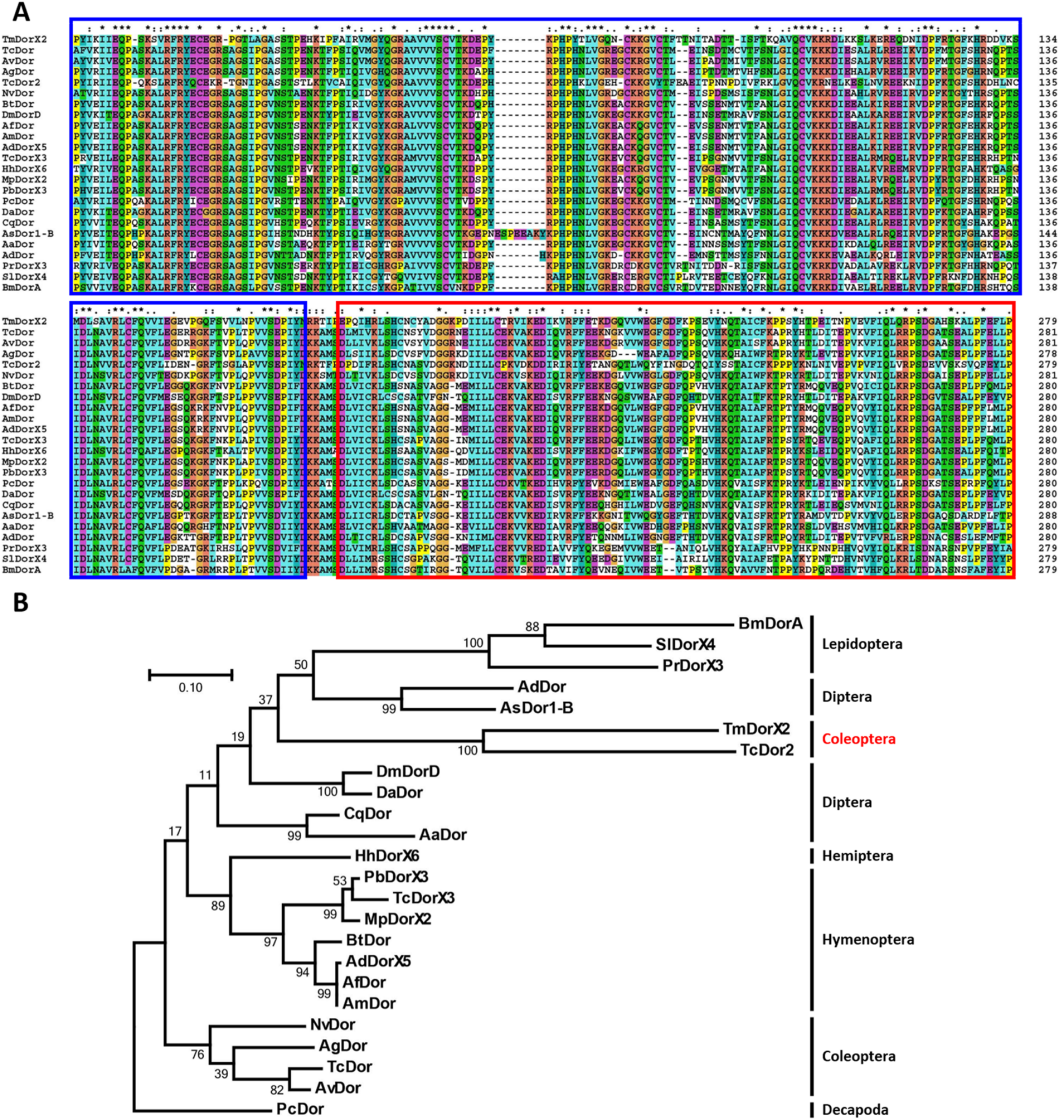
Multiple sequence alignment of *TmDorX2* with its orthologues (**A**) and phylogenetic analysis (**B**). The sequences of representative Dorsal proteins showed high homology at the conserved Rel homology domains (RHD) marked in blue boxes and the immunoglobulin-like, plexins, transcription factors (IPT) domains marked in red boxes. The symbols indicate conservation scores between groups according to the Gonnet PAM 250 matrix (‘*’>‘:’>‘.’) and ‘−’ indicates internal or terminal gaps (**A**). The phylogenetic tree was constructed using the maximum likelihood method and bootstrapped 1,000 times in the MEGA 7.0 program based on the multiple alignment by ClustalX2.1. Protein sequences used in this study are as follows: *Tm*DorX2 (*Tenebrio molitor* Dorsal protein isoform X2), *Dm*DorD (*Drosophila melanogaster* Dorsal, isoform D; NP_001163000.1), *Aa*Dor (*Aedes albopictus* embryonic polarity protein dorsal-like; XP_019931750.1), *Ag*Dor; (*Anopheles glabripennis* embryonic polarity protein dorsal-like isoform X3; XP_018572644.1), *Af*Dor (*Apis florea*; embryonic polarity protein dorsal-like isoform X3; XP_012343274.1), *Am*Dor (*Apis mellifera* dorsal protein isoform X2; XP_006567065.1), *Ad*DorX5 (*Apis dorsata* embryonic polarity protein dorsal-like isoform X5; XP_006619742.1), *Bt*Dor (*Bombus terrestris* embryonic polarity protein dorsal isoform X2; XP_012174122.1), *Nv*Dor (*Nicrophorus vespilloides* putative transcription factor p65 homolog isoform X2; XP_017781152.1), *Av*Dor (*Asbolus verrucosus* dorsal, partial; RZB54393.1), *Tc*Dor (*Tribolium castaneum* Dorsal; EFA02850.1), *Tc*Dor2 (*Tribolium castaneum* Dorsal 2; EFA02885.1), *Tc*DorX3 (*Trachymyrmex cornetzi* embryonic polarity protein dorsal isoform X3; XP_018357420.1), *Hh*DorX6 (*Halyomorpha halys* embryonic polarity protein dorsal-like isoform X6; XP_014275495.1), *Pb*DorX3 (*Pogonomyrmex barbatus* embryonic polarity protein dorsal isoform X3; XP_006567063.1), *Mp*DorX2 (*Monomorium pharaonis* embryonic polarity protein dorsal isoform X2; XP_011647341.1), *Da*Dor (*Delia antiqua* Dorsal; AFI98401.1), *Ad*Dor (*Anopheles darlingi* Rel1/Dif/Dorsal; ETN66814.1), *Bm*DorA (*Bombyx mori* embryonic polarity protein dorsal isoform A; NP_001166296.1), *Sl*DorX4 (*Spodoptera litura* embryonic polarity protein dorsal isoform X4; XP_022815079.1), *Pr*DorX3 (*Pieris rapae* embryonic polarity protein dorsal isoform X3; XP_022116742.1), *Cq*Dor (*Culex quinquefasciatus* embryonic polarity protein dorsal; XP_001844078.1), and *As*Dor1-B (*Anopheles sinensis* Dorsal isoform 1-B; KFB39849.1). *Pc*Dor *(Penaeus chinensis* dorsal; ACJ36225.1) was used as an outgroup (**B**).

A local tblastn search using the *T. castaneum* Dorsal 2 protein sequence (GenBank: EFA02885.1) as the query and the *T. molitor* RNAseq and EST libraries as the subject was sufficient to identify the Dorsal homologue from *T. molitor* (Designated as *TmDorX2*; accession number: MN056348). The genomic organization of *TmDorX*2 showed that it contains five exons and four introns (Fig. S2). The *Tm*DorX2 full-length ORF consists of 1,482 bp, encoding a polypeptide of 493 amino acid residues (Fig. 1). According to InterProScan analysis and the NCBI conserved domain database, the *Tm*DorX2 amino acid sequence comprises a RHD (P_24_ to N_193_), an IPT (E_199_ to P_302_), and a nuclear localization signal (NLS; P_307_GALKRKREKY_317_). Four putative NF-κB signature sequences (I_28_ to P_46_; G_215_ to F_235_; F_251_ to Y_269_; H_292_ to K_306_) were found at the N-terminus of the RHD and the N- and C-terminusof the IPT domain. To evaluate the evolutionary position of *Tm*DorX2 using percentage identity and phylogenetic analysis, we retrieved orthologous sequences from 22 insect species. Furthermore, the conserved RHD and IPT domain in *Tm*DorX2 were compared at the amino acid level using ClustalX 2.1 multiple sequence alignment (Fig. 2A).

Phylogenetic analysis revealed that the Dorsal isoforms from representative insect species clustered under separate insect orders (Fig. 2B). The Dorsal X2 isoforms from *T. molitor* and *T. castaneum* were clustered together, as confirmed by bootstrap replications. The Dorsal isoforms from the order Coleoptera were clustered separately. Similarly, the Dorsal isoforms from insect orders Lepidoptera, Hemiptera and Hymenoptera were classified into separate independent clusters. Moreover, species belonging to the order Hymenoptera (including ants and bees) formed two distinct clusters, one formed by ants [*Pb*DorX3 (*Pogonomyrmex barbatus*), *Tc*DorX3 (*Trachymyrmex cornetzi*), and *Mp*DorX2 (*Monomorium pharaonis*)] and another by bees [*Bt*Dor (*Bombus terrestris*), *Ad*DorX5 (*Apis dorsata*), *Af*Dor (*Apis florea*), and *Am*Dor (*A. mellifera*)]. Whereas *Drosophila* Dorsal isoforms showed relatedness, the Dorsal isoforms belonging to other species in the order Diptera were clustered separately.

Percent identity calculated based on specific domain analysis showed that *TmDorX2* has the highest similarity with *Tc*Dor and *Av*Dor (64% identity), followed by 59% and 57% identity with *Tc*Dor2 and *Nv*Dor, respectively. In addition, the maximum and minimum identities of *TmDorX2* within the Hymenoptera (56–57%), Diptera (51–54%), and Lepidoptera (46–49%) orders were calculated and are presented in Fig. S3.

### Temporal and spatial expression patterns of *TmDorX2*

**Figure 3:**
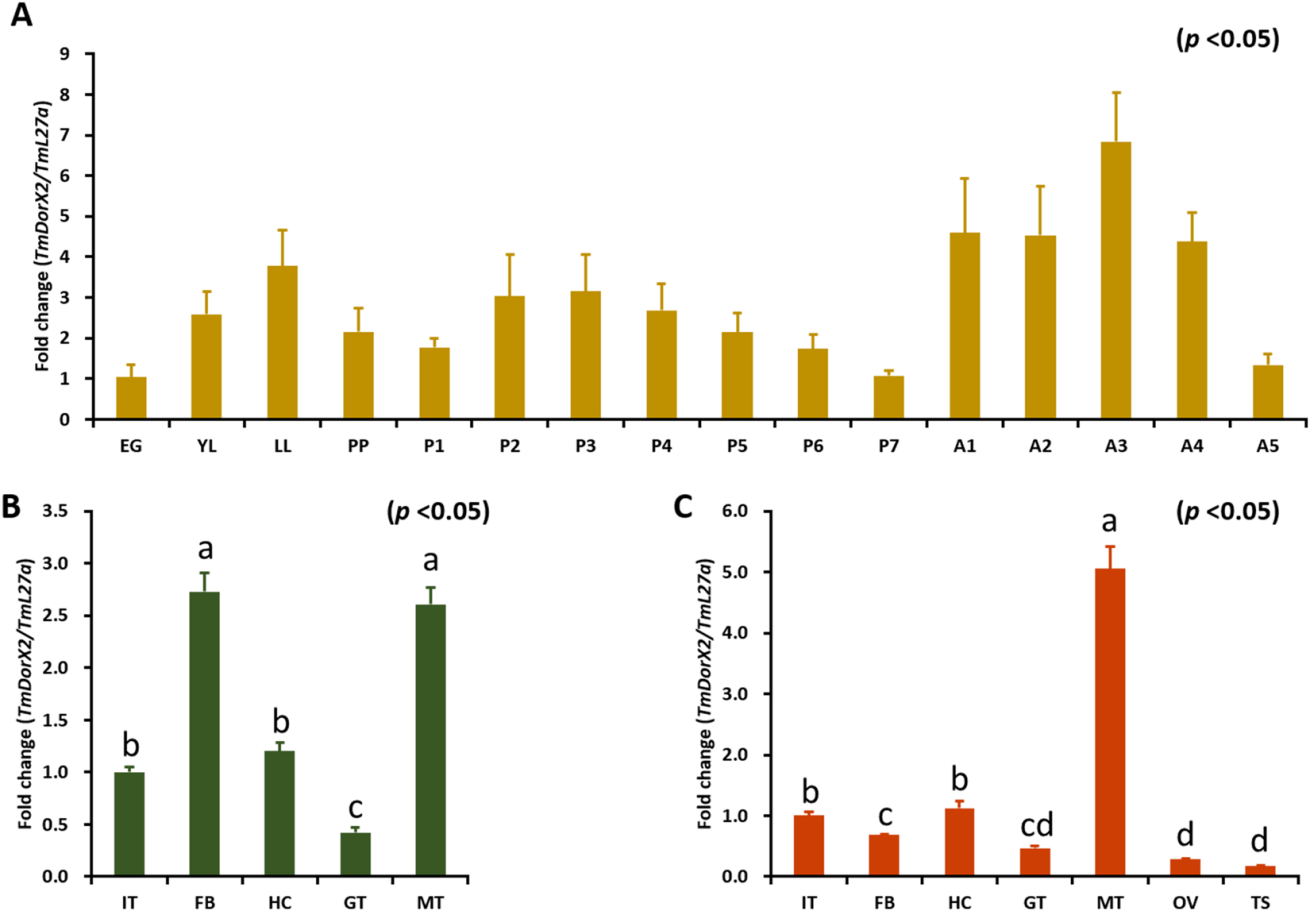
Relative *TmDorX2* mRNA levels during development (**A**) and in different tissues of *T. molitor* late-instar larvae (**B**) and 5-day-old adults (**C**) measured by qRT-PCR. (**A**) *TmDorX2* expression levels are shown for eggs (EG), young larvae (YL), late-instar larvae (LL), Pre-pupa (PP), 1 to 7-day-old pupa (P1-P7), and 1 to 5-day-old adults (A1-A5). (**B**) The expression levels of *TmDorX2* in the integument (IT), fat body (FB), hemocytes (HC), gut (GT), and Malpighian tubules (MT) are shown for late-instar larvae. In addition to the above tissues, the expression levels of *TmDorX2* in the ovary (OV) and testis (TS) is depicted in 5-day-old adults (**C**). The Y-axis represents the relative expression level of *TmDorX2*. The *T. molitor* 60 S ribosomal protein L27a (*TmL27a*) served as an endogenous control to normalize RNA levels between samples. Vertical bars indicate mean ± SE (n = 20).

qRT-PCR was employed to investigate *TmDorX2* mRNA expression during different developmental stages of the insect relative to the levels of *TmL27a* as an internal control (Fig. 3A). The *TmDorX2* mRNA was expressed during all developmental stages of *T. molitor*. The mRNA levels were upregulated in the late larval stage, followed by a decline in expression in early pupae. This was followed by an increase in mRNA expression in 2-day old pupae and a consistent decline in late pupal stages. The expression of *TmDorX2* was higher during adult stages, with the highest expression observed in 3-day-old adults (Fig. 3A). The *TmDorX2* mRNA was detected in all *T. molitor* larval tissues with the highest expression level observed in the fat body and MTs, followed by that in the integuments and hemocytes, while the lowest expression was observed in the gut (Fig. 3B). Likewise, the transcription of *TmDorX2* in 5-old-day adults was significantly higher in MTs, followed by that in the integument and hemocytes. The lowest expression of *TmDorX2* was found in reproductive tissues (Fig. 3C).

### Induction profile of *TmDorX2* upon microbial insult

**Figure 4:**
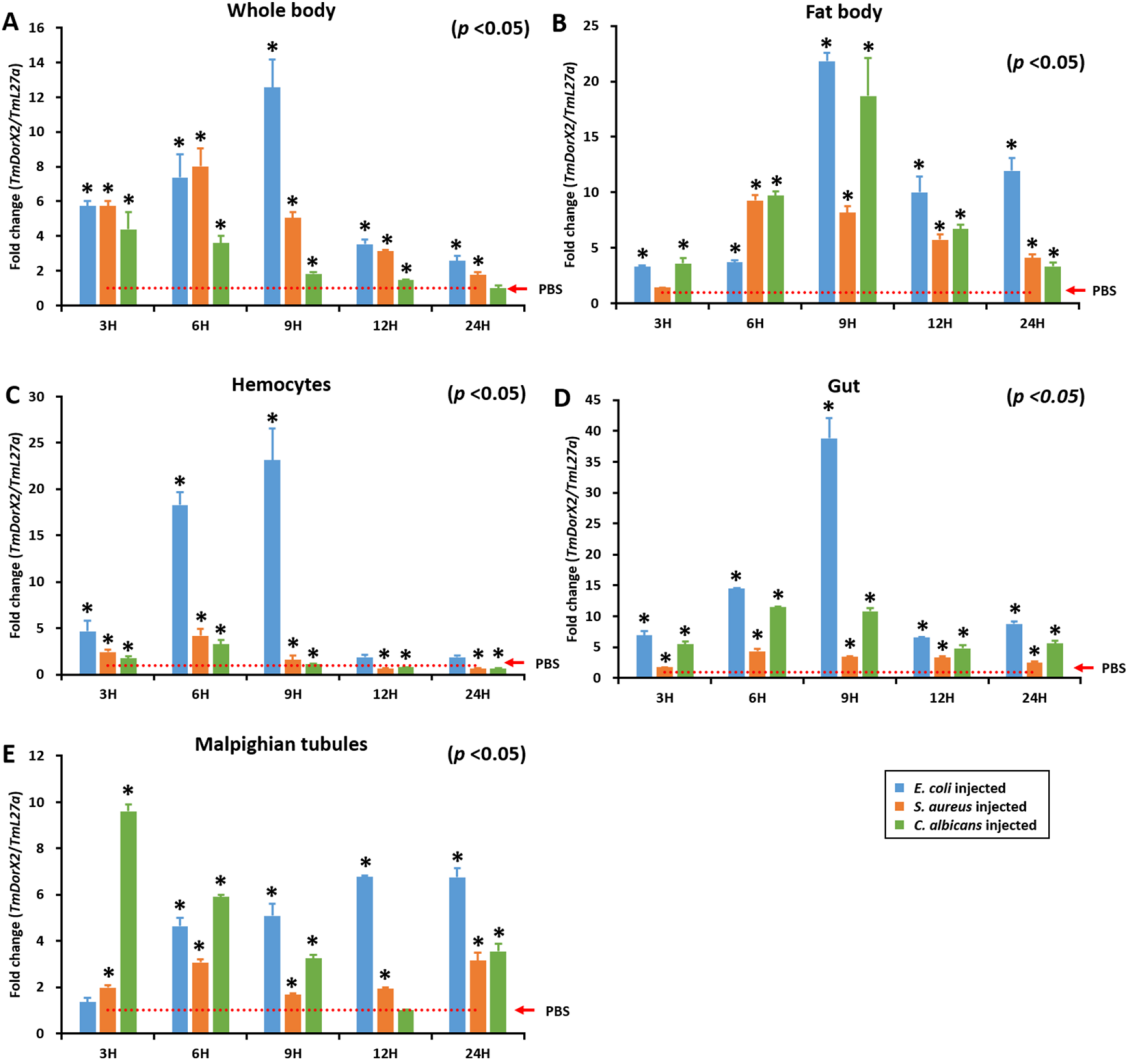
Temporal expression profiles of *TmDorX2* in response to *E. coli*, *S. aureus*, and *C. albicans* infection in the whole body (**A**), fat body (**B**), hemocytes (**C**), gut (**D**), and Malpighian tubules (**E**) of *T. molitor* young larvae (10^th^ to 12^th^ instar larvae), examined by qRT-PCR at 3, 6, 9, 12, at 24 h post-infection. *TmDorX2* expression levels were normalized to their levels in phosphate buffered saline (PBS)-injected controls. The *T. molitor* 60 S ribosomal protein L27a (*TmL27a*) was used as an internal control. Vertical bars indicate mean ± SE (n = 20). ‘*’ Indicates significant differences (*p* < 0.05).

To elucidate the participation of *TmDorX2* in *T. molitor* innate immunity upon microbial infection, we evaluated the levels of *TmDorX2* mRNA expression by qRT-PCR at different time points (3, 6, 9, 12, and 24 h) in the fat body, hemocytes, gut, MTs, and the whole body of *T. molitor* larvae (Fig. 4). In *Tenebrio* larvae injected with Gram-negative bacteria *E. coli*, the whole-body levels of *TmDorX2* expression increased 9 h post-injection (hpi), followed by a gradual decline at 12 hpi and 48 hpi (Fig. 4A). Significant increase in whole-body *TmDorX2* expression was also observed after *S. aureus* infection, at all time points tested, when compared with that in the mock control (*p* < 0.05). In the fat body tissue, the fold-increase in *TmDorX*2 expression upon infection with *E. coli* and the fungus *C. albicans* reached its highest level at 9 hpi. *S. aureus* infection also caused a significant increase in *TmDorX2* expression in the fat body when compared with that in the mock-infected larvae (*p* < 0.05) (Fig. 4B). In hemocytes, *E. coli* infection led to a dramatic increase in *TmDorX2* expression at early stages of infection followed by a large decline at later time points (Fig. 4C). Similar *TmDorX2* expression profiles were observed in the gut after *E. coli* infection. The increase in *TmDorX2* expression in the gut was significant when compared with the mock control (*p* < 0.05) (Fig. 4D). In the MTs, an early increase in the expression of *TmDorX2* mRNA was observed after *C. albicans* infection, which was statistically significant (*p* < 0.05) (Fig. 4E).

### Mortality upon *TmDorX2* knockdown and microbial challenge

**Figure 5:**
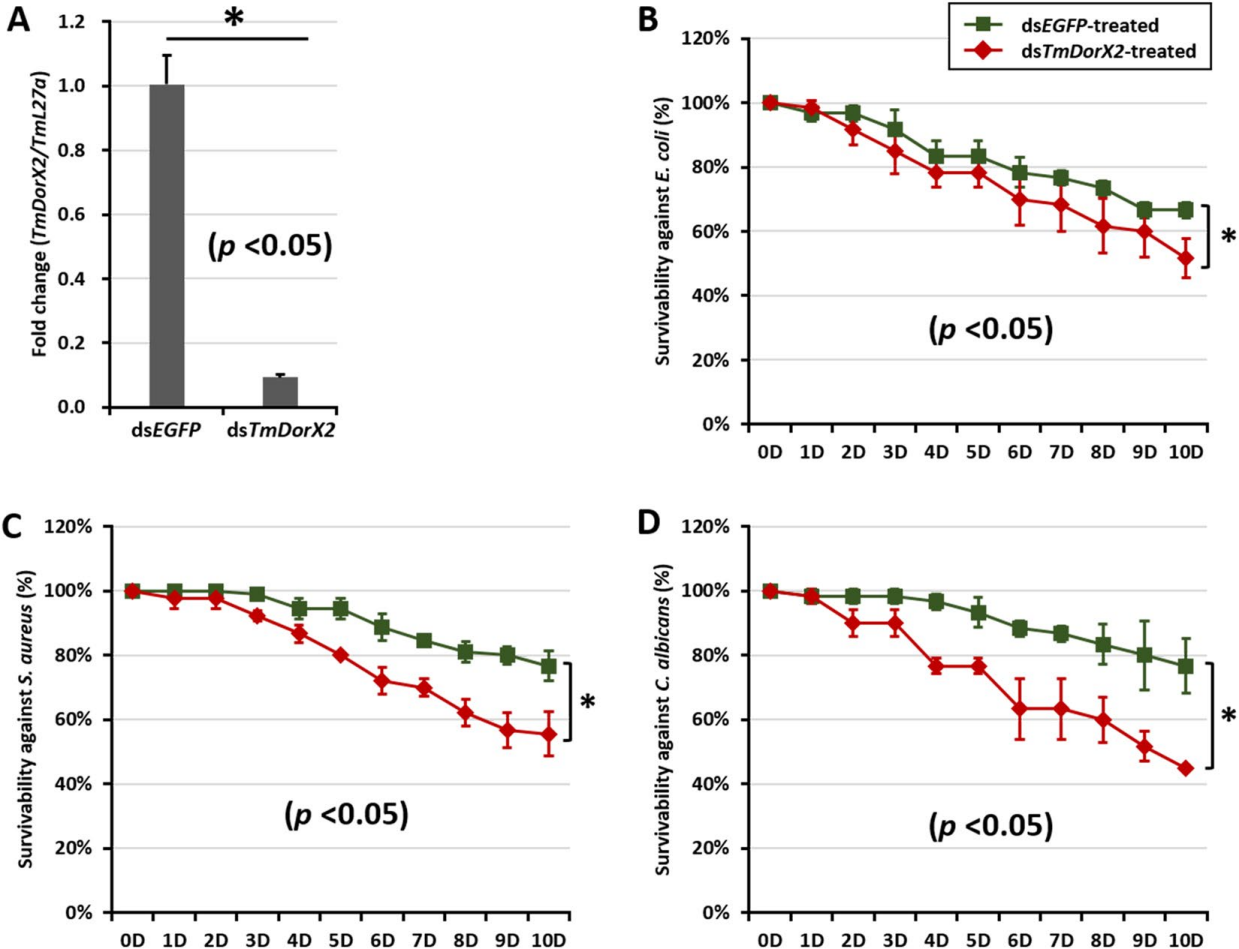
Effect of *TmDorX2* dsRNA (RNAi) on the survival of *T. molitor* larvae (n = 10 per group), observed for 10 days after microbial insult. (**A**) Silencing efficiency of *TmDorX2* RNAi assessed by measuring the *TmDorX2* mRNA levels by qRT-PCR 2 days post-injection. Survival of *T. molitor* larvae injected with ds*TmDorX2* after immune challenge with (**B**) *E. coli* (**C**) *S. aureus*, and (**D**) *C. albicans*. ds*EGFP* injected groups acted as negative control. The data are an average of three independent biological replicate experiments. ‘*’ Represents significant differences between ds*TmDorX2-* and ds*EGFP*-treated groups (*p* < 0.05).

Double-stranded RNA (dsRNA) against *TmDorX2* was synthesized and used to assess the involvement of *TmDorX2* in the Toll pathway. Subsequently, the transcription of 14 AMP genes was examined to determine their regulation by the Toll signaling pathway. Evaluation of the levels of *TmDorX2* mRNA expression after dsRNA injection in comparison to those in the ds*EGFP* treated negative control demonstrated a 91% knockdown efficiency for *TmDorX2*. (Fig. 5A). To examine the effect of *TmDorX2* silencing on larval survival after challenging with *E. coli*, *S. aureus*, and *C. albicans*, we performed experiments in two sets of *T. molitor* larvae treated with ds*TmDorX2* and ds*EGFP*, respectively, and counted the dead insects for 10 days. Larval mortality was compared between the ds*EGFP-* and ds*TmDorX2-*treated groups and a difference at *p* < 0.05 was considered significant. The results show that the mortality rate resulting from *E. coli* (48%, Fig. 5B) and *S. aureus* (44%, Fig. 5C) infections in the ds*TmDorX2*-treated groups were significantly different from that in the corresponding ds*EGFP* groups, respectively. The percent mortality of *C. albicans* challenged larvae was 55%, and was significantly different than that of the ds*EGFP*-treated larvae (*p* < 0.05) (Fig. 5D).

### Role of *TmDorX2* in *T. molitor* AMP gene expression

Given that the stimulation of immune signaling cascades leads to the production of antibacterial or antifungal AMPs to combat the invading pathogens, we investigated the expression of 14 *T. molitor* AMP genes, namely *TmTene1*, *−2*, *−*3, and *−4*, *TmAtt1a*, *−1b*, and *−2*, *TmDef1* and *−2*, *TmCole1* and *−2*, *TmCec2*, and *TmTLP1* and *−2*. We hypothesized that the significant mortality observed in the ds*TmDorX2*-treated group after microbial challenge was due to the downregulation of AMP gene expression. To confirm our hypothesis, experiments were performed with two groups of *T. molitor* larvae, injected with 1 μl (1 μg) of *TmDorX2* dsRNA and ds*EGFP*, respectively. After confirming *TmDorX2* silencing 2 days after dsRNA injection, larvae were injected with *E. coli*, *S. aureus*, *C. albicans*, or PBS. One-day post-microbial infection, the AMP gene expression profile was studied by qRT-PCR in dissected immune tissues, such as the fat body, hemocytes, gut, and MTs.

**Figure 6:**
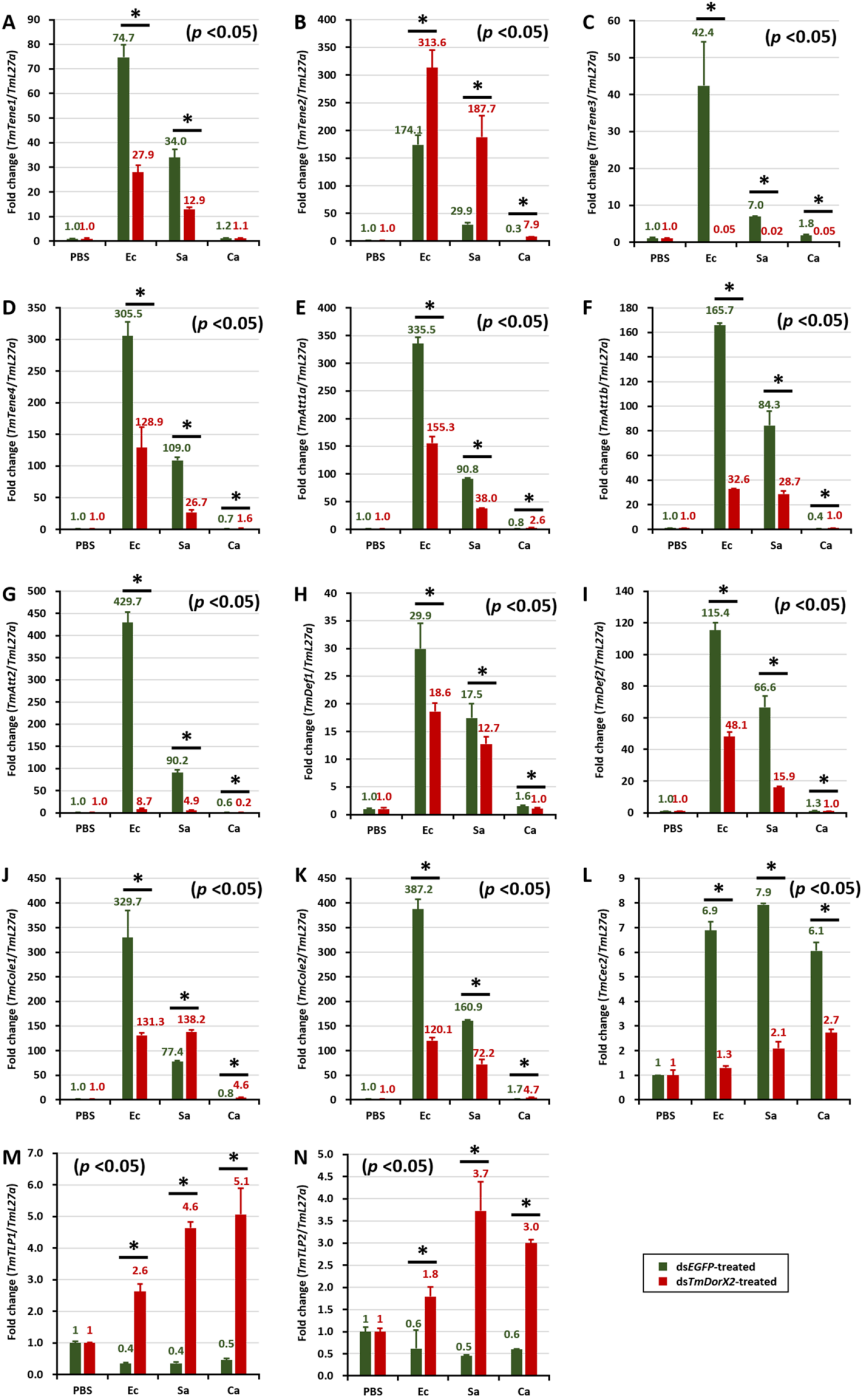
Antimicrobial peptide (AMP) induction patterns in *TmDorX2*-silenced *T. molitor* larval fat body in response to *E. coli* (Ec)*, S. aureus* (Sa), or *C. albicans* (Ca) infection. Gram-negative bacteria (*E. coli*), Gram-positive bacteria (*S. aureus*), and fungi (*C. albicans*) were injected into ds*TmDorX2*-treated *T. molitor* larvae on the second day post-dsRNA injection, the larvae were dissected 24 h post-microbial infection. The expression levels of the following AMP genes were analyzed by qRT-PCR: *TmTenecin-1* (*TmTene1*, **A**); *TmTenecin-2* (*TmTene2*, **B**); *TmTenecin-3* (*TmTene3*, **C**); *TmTenecin-4* (*TmTene4*, **D**); *TmAttacin-1a* (*TmAtt1a*, **E**); *TmAttacin-1b* (*TmAtt1b*, **F**); *TmAttacin-2* (*TmAtt2*, **G**); *TmDefensin-1* (*TmDef1*, **H**); *TmDefensin-2* (*TmDef2*, **I**);*TmColeptericin-1* (*TmCole1*, **J**); *TmColeptericin-2* (*TmCole2*, **K**); *TmCecropin-2* (*TmCec2*, **L**); *TmTLP-1* (*TmTLP1*, **M**); and *TmTLP-2* (*TmTLP2*, **N**) ds*EGFP* was used as a negative control and *TmL27a* served as an internal control. The numbers above the bars represent the AMP transcription levels. All experiments were repeated three times with similar results.

**Figure 7:**
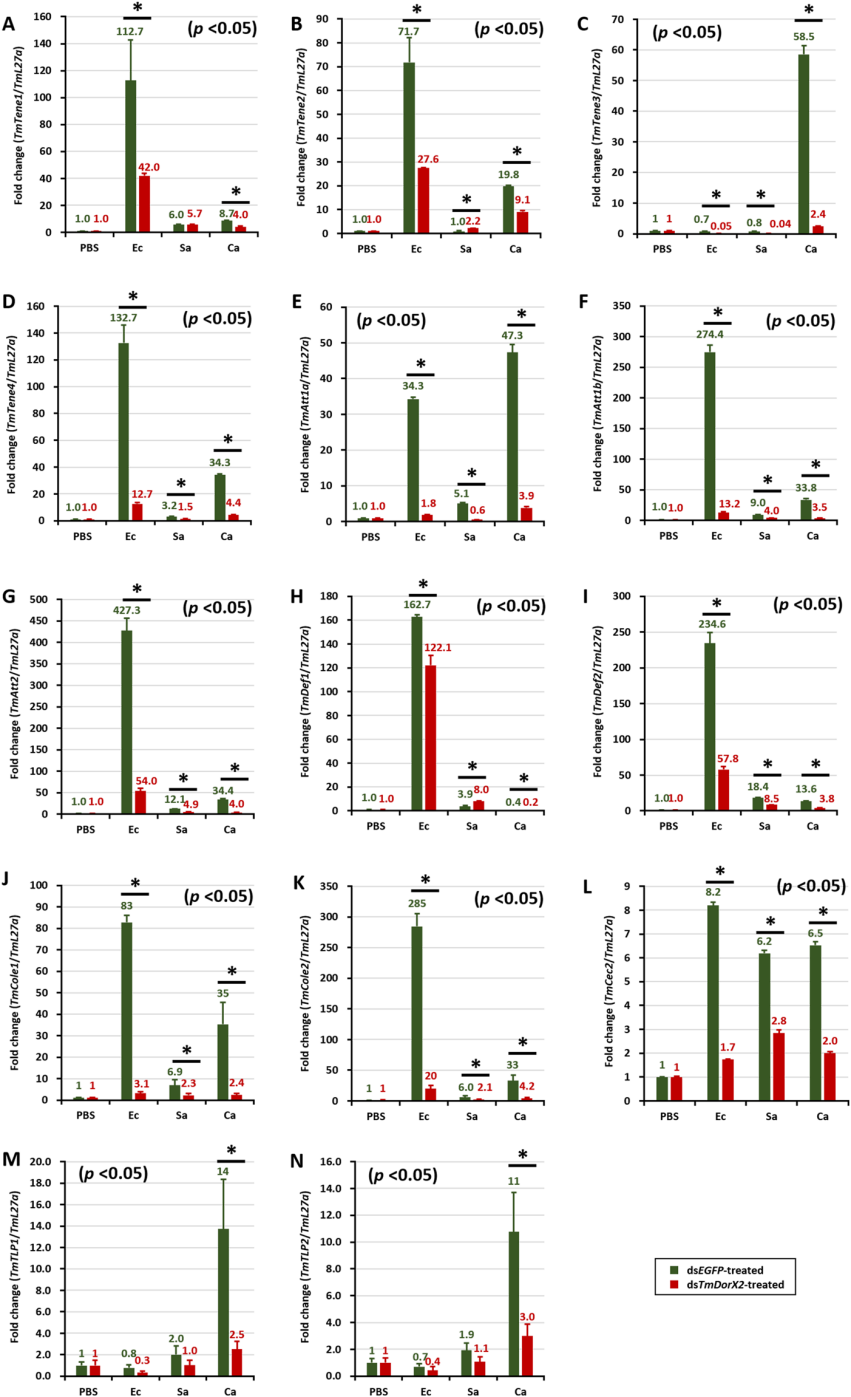
Effect of ds*TmDorX2* on antimicrobial peptide (AMP) gene expression in *T. molitor* larval gut infected with *E. coli* (Ec), *S. aureus* (Sa), and *C. albicans* (Ca). On the second day after dsRNA *TmDorX2* injection, at which point the mRNA levels of *TmDorX2* were reduced by 91% in the ds*TmDorX2*-treated groups compared to the ds*EGFP-*treated groups, the larvae were exposed to the microbes. The expression levels of 14 AMP genes were analyzed by qRT-PCR: *TmTene1* (**A**); *TmTene2* (**B**); *TmTene3* (**C**); *TmTene4* (**D**); *TmAtt1a* (**E**); *TmAtt1b* (**F**); *TmAtt2* (**G**); *TmDef1* (**H**); *TmDef2* (**I**); *TmCole1* (**J**); *TmCole2* (**K**); *TmCec2* (**L**); *TmTLP-1* (**M**); and *TmTLP-2* (**N**). ds*EGFP* was used as a negative control and *TmL27a* served as an internal control. The numbers above the bars show the transcript expression levels. Statistical analysis was performed using Student’s t-tests (*p* < 0.05).

In the fat body of *TmDorX2*-silenced larvae, the expression of 11 AMP genes namely *TmTene1, −3* and *−4* (Fig. 6A,C,D), *TmAtt1a*, *−1b* and *−*2 (Fig. 6E–G), *TmDef1* and *−*2 (Fig. 6H,I), *TmCole1*, and *−*2 (Fig. 6J,K), and *TmCec2* (Fig. 6L), was noticeably decreased upon *E. coli* challenge (Fig. 6). In contrast, the transcription of *TmTene*2 (Fig. 6B), *TmTLP1* (Fig. 6M), and *TmTLP2* (Fig. 6N) was upregulated in ds*TmDorX2*-injected larvae upon *E. coli, S. aureus*, or *C. albicans* challenge, as compared to that in ds*EGFP* injected larvae. Simultaneously, there was a considerable decline in the expression of the same 11 AMP genes, as well as in the *TmTene2* mRNA level, in the gut (Fig. 7). Furthermore, *TmDorX2* knockdown reduced the expression of 8 AMPs in hemocytes and MTs of *E. coli* challenged larvae. This suggests that the downregulation of AMP gene expression in the fat body and gut of ds*TmDorX2* larvae after *E. coli* infection is more striking than the reduction of AMP genes in hemocytes and MTs. Upon *S. aureus* infection, the transcription of 10 AMPs, namely *TmTene1*, *−3*, and *−4* (Fig. 6A,C,D), *TmAtt1a*, *−1b* and *−2* (Fig. 6E–G), *TmDef1* and *−2* (Fig. 6H,I), *TmCole2* (Fig. 6K), and *TmCec2* (Fig. 6L), was upregulated in the fat body of ds*EGFP*-treated groups, whereas it was decreased in ds*TmDorX2*-injected groups, suggesting that the fat body is a crucial tissue for combatting *S. aureus* infection. In addition to changes in the fat body, *S. aureus* infection caused a significant downregulation of AMPs *TmTene1* (Fig. 7A), *TmAtt1a*, *1b*, and *2* (Fig. 7E–G), *TmDef1* and *2* (Fig. 7H,I), *TmCole1* and 2 (Fig. 7J,K) in hemocytes as well.

**Figure 8:**
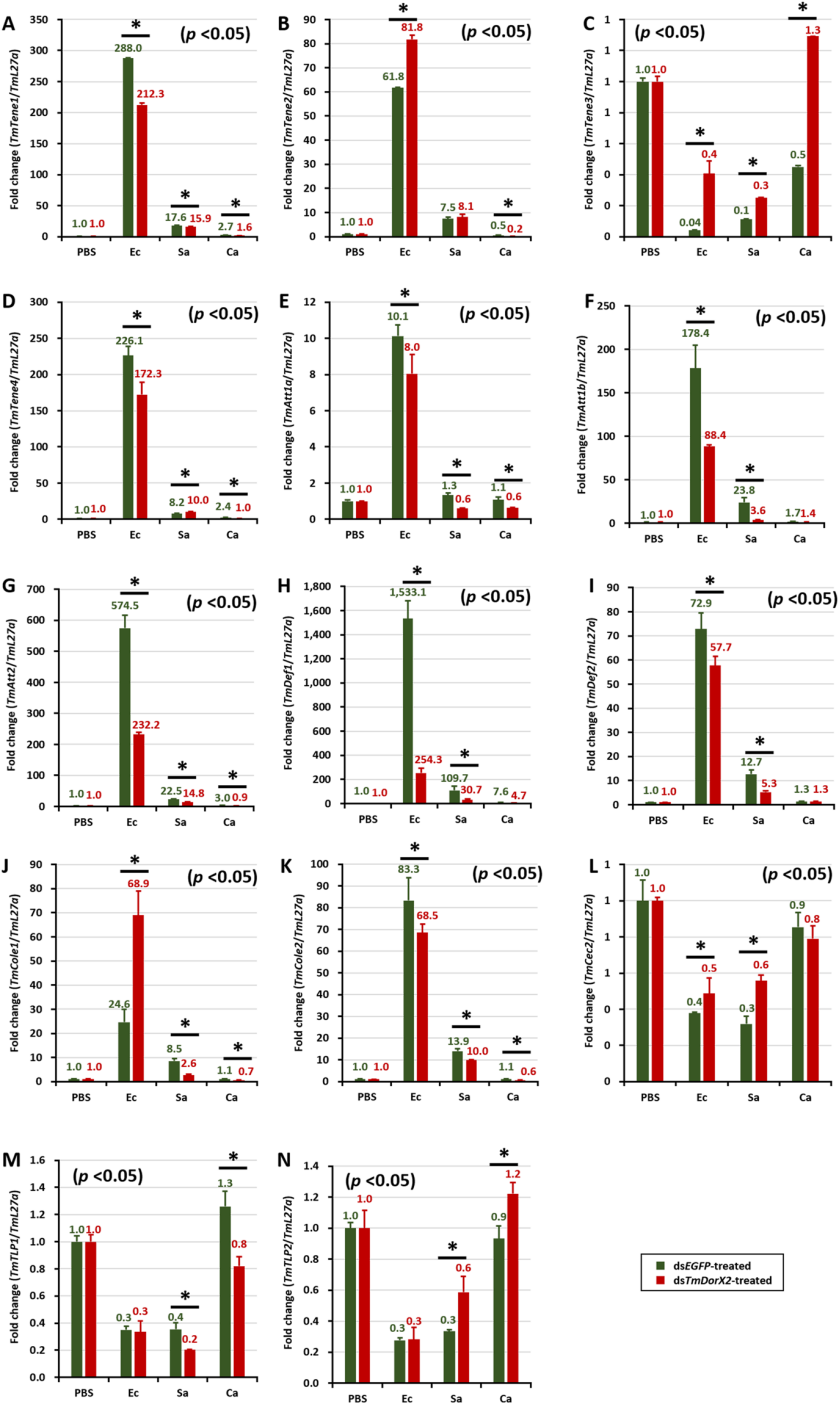
Expression levels of antimicrobial peptides (AMPs) in *TmDorX2-*silenced *T. molitor* hemocytes upon *E. coli*, *S. aureus*, or *C. albicans* infection. The qRT-PCR expression profiles of *TmTene1* (**A**), *TmTene-2* (**B**), *TmTene3* (**C**), *TmTene4* (**D**), *TmDef1* (**E**), *TmDef2* (**F**), *TmCec2* (**G**), *TmCole1* (**H**), *TmCole2* (**I**), *TmAtt1a* (**J**), *TmAtt1b* (**K**), *TmAtt2* (**L**), *TmTLP1* (**M**), and *TmTLP2* (**N**) are shown. ds*EGFP* was used as a negative control and *TmL27a* was used as an internal control. All experiments were performed at least three times, and statistical analysis was performed using Student’s t-tests (*p* < 0.05).

Intriguingly, the expression of *TmTene2, −3* and *−4* (Fig. 7B–D), *TmAtt1a*, *−1b*, and *−*2 (Fig. 7E–G), *TmCole1* and *−*2 (Fig. 7J,K), *TmCec2* (Fig. 7L), and *TmTLP1* and *−*2 (Fig. 8M,N) in the gut of ds*EGFP*-treated groups were upregulated by *C. albicans* and bacterial infection, whereas it declined markedly in ds*TmDorX*2-treated larvae. In addition, levels of the antifungal AMPs *TmTene3, TmTLP1* and *TmTLP*2 (Fig. 8C,M,N) were noticeably higher in ds*EGFP* groups and lower in the ds*TmDorX2*-treated groups upon *C. albicans* infection, suggesting that *TmDorX2* specifically regulates antifungal AMPs in the gut in response to *C. albicans*.

**Figure 9:**
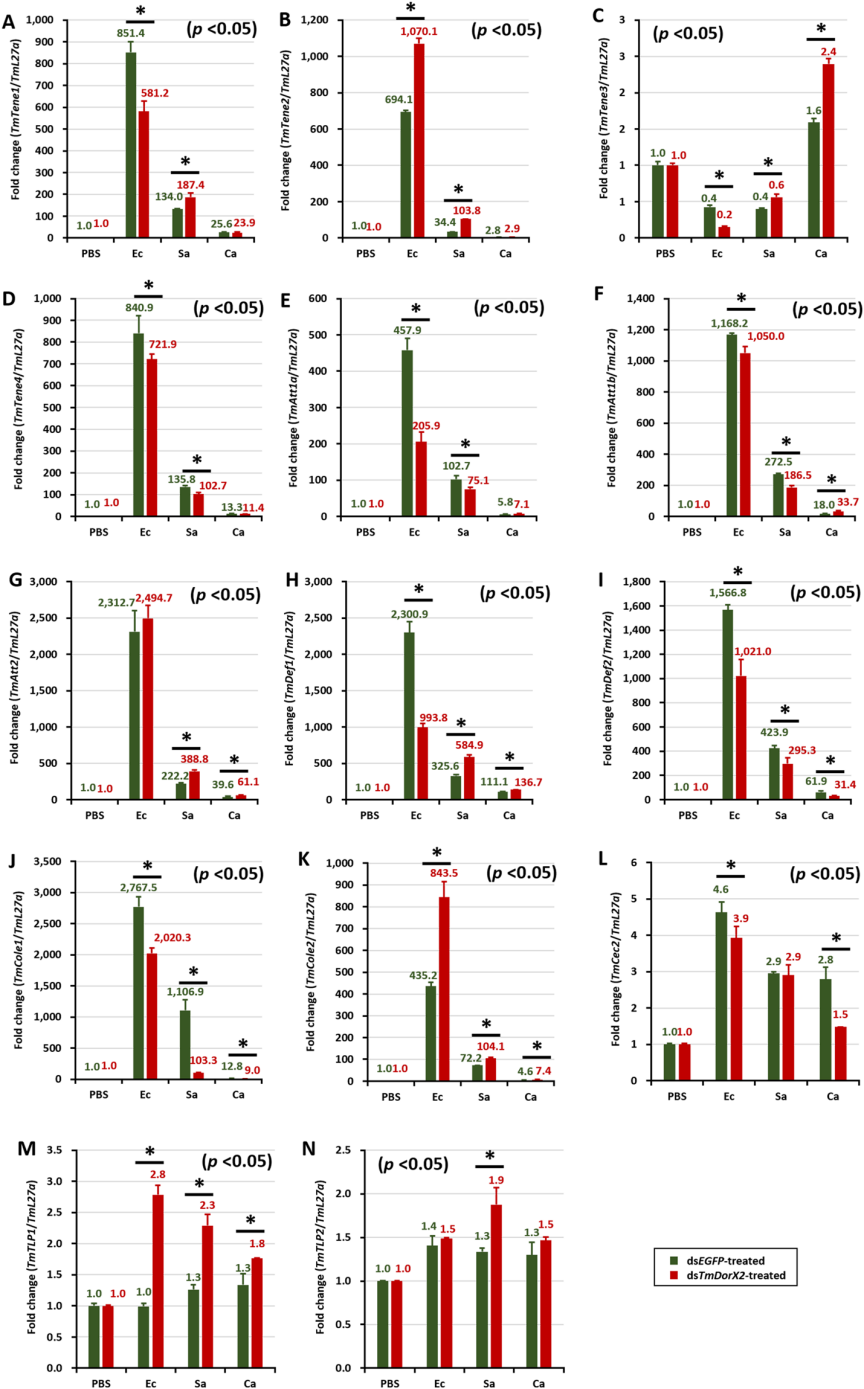
Antimicrobial peptide (AMP) expression levels in *TmDorX2*-knockdown *T. molitor* larval MTs upon *E. coli* (Ec), *S. aureus* (Sa), or *C. albicans* (Ca) infection on the second day post-*TmDorX2* silencing. Individuals from each group were dissected 24 h after microbial challenge. The expression levels of the following AMPs were measured by qRT-PCR:*TmTenecin-1* (*TmTene1*, **A**); *TmTenecin-2* (*TmTene2*, **B**); *TmTenecin-3* (*TmTene3*, **C**); *TmTenecin-4* (*TmTene4*, **D**); *TmAttacin-1a* (*TmAtt1a*, **E**); *TmAttacin-1b* (*TmAtt1b*, **F**); *TmAttacin-2* (*TmAtt2*, **G**);*TmDefensin-1* (*TmDef1*, **H**); *TmDefensin-2* (*TmDef2*, **I**); *TmColeptericin-1* (*TmCole1*, **J**); *TmColeptericin-2* (*TmCole2*, **K**); *TmCecropin-2* (*TmCec2*, **L**); *TmTLP-1* (*TmTLP1*, **M**); and *TmTLP2* (*TmTLP-2*, **N**). ds*EGFP* was used as a negative control and *TmL27a* was used as an internal control. The numbers above the bars indicate AMP mRNA expression levels. All experiments were repeated three times with similar results.

**Figure 10:**
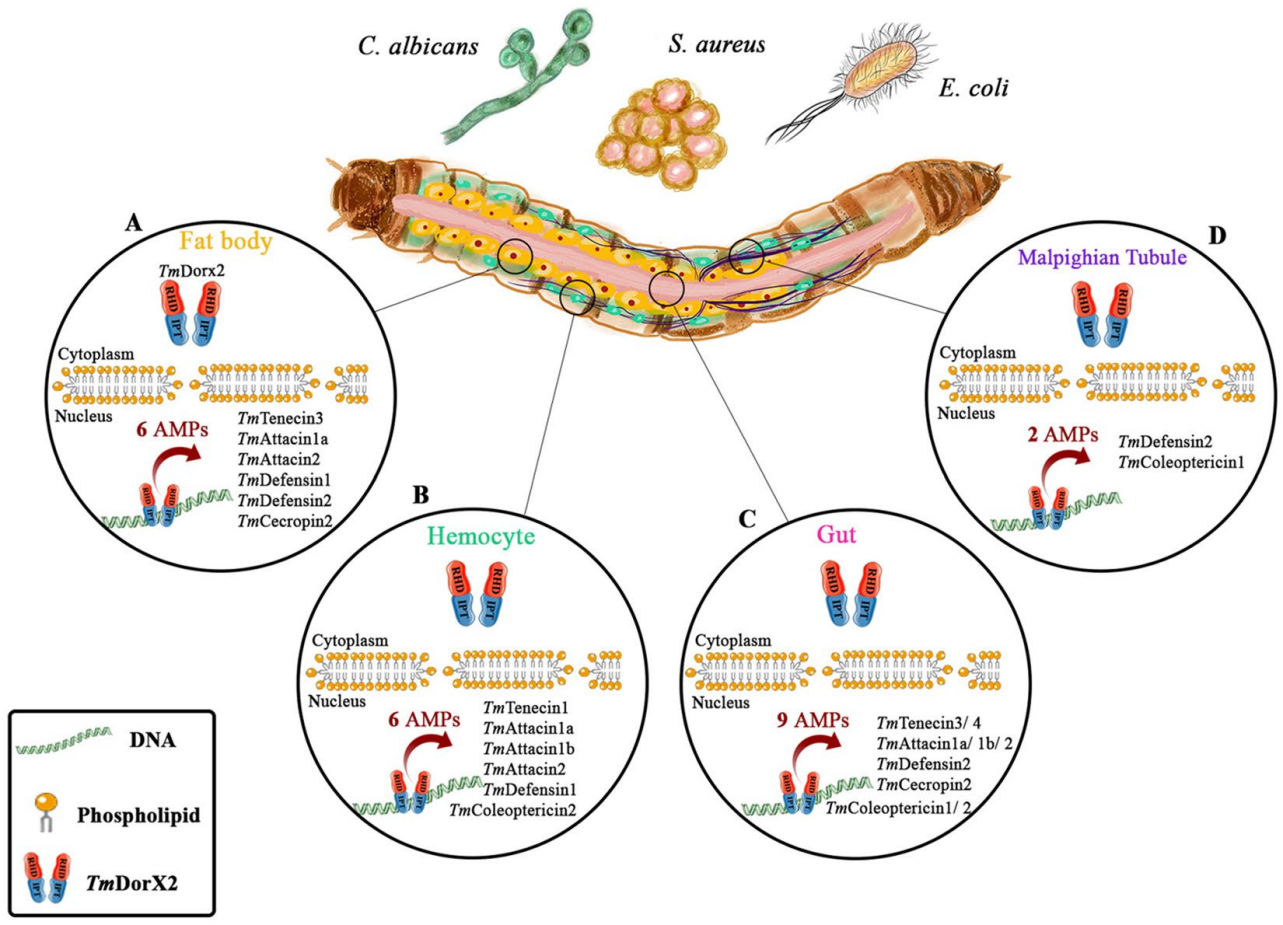
A schematic representation of the positive role of *TmDorX2*(downstream of Toll pathway) in controlling antimicrobial peptide (AMP) expression in the fat body (**A**), hemocytes (**B**)¸ gut (**C**), and Malpighian Tubules (**D**) of young larvae infected with *E. coli*, *S. aureus*, and *C. albicans*. In the fat body, 6 AMP-encoding genes are regulated by *TmDorX2* upon fungal and bacterial infections (**A**). *TmDorX2* affects the expression of *TmTenecin-1*, *TmAttacin-1a*, -*1b*, and -*2*, *TmDefensin-1*, and *TmColeoptericin-2* in hemocytes in response to *E. coli*, *S. aureus*, and *C. albicans* infection. In the gut, 9 AMPs are downregulated in *TmDorX2*-silenced larvae, suggesting that *TmDorX2* positively controls the induction of these genes (**C**). In the Malpighian Tubules, only 2 AMP-encoding genes, namely *TmDefensin-2* and *TmColeoptericin-1* are regulated by the *Dorsal* in *T. molitor* after microbial infection (**D**).

In MTs, the requirement for *TmDorX2* against *C. albicans* challenge was less marked as compared to that in the gut tissue. As shown in Figs 9 and 10, AMPs, such as *TmTene1, TmTene*4*, TmAtt1a, TmDef1, TmDef2* and *TmCole1*, were downregulated in ds*TmDorX2*- injected larvae challenged with *E. coli*, *S. aureus*, and *C. albicans*. Moreover, *TmTene*4 (Fig. 9D), *TmAtt1a*, *1b* (Fig. 9E,F), *TmDef*2 (Fig. 9I), and *TmCole1* (Fig. 9J) showed decreased expression in *TmDorX*2-depleted groups compared to controls upon *S. aureus* challenge, similar to other tissues. However, *S. aureus* induced the expression of *TmTene1, TmTene2* (Fig. 9A,B)*, TmAtt2* (Fig. 9G)*, TmDef1* (Fig. 9H), *TmCole2* (Fig. 9K)*, TmTLP1*, and *TmTLP2* (Fig. 9M,N) in ds*TmDorX2*-treated larvae. During *C. albicans*, similar to *S. aureus*, the mRNA levels of *TmAtt2* (Fig. 9G), *TmDef1* (Fig. 9H), *TmCole2* (Fig. 9K)*, TmTLP1*, and *TmTLP2* (Fig. 9M,N) were increased in the *TmDorX2*-silenced groups, whereas the expression of *TmDef2* (Fig. 9I) and *TmCole1* (Fig. 9J) was decreased.

In general, after infection with all chosen microbes (*E. coli*, *S. aureus*, and *C. albicans*), the expression of several AMP genes was significantly decreased in *TmDorX2*-silenced larvae in comparison with ds*EGFP*-injected cohorts. Our present data demonstrate that *TmAtt1a* (Figs 7E and 8E) and *TmAtt 2* (Figs 7G and 8G) were downregulated in hemocytes and the gut of *TmDorX2*-silenced larvae. Moreover, *TmTene3* (Figs 6C and 8C), *TmDef2* (Figs 6I, 8I and 9I) and *TmCec2* (Figs 6L and 8L) were highly downregulated in the fat body, gut and MTs of ds*TmDorX2* groups. Finally, in the hemocytes and gut, expression of *TmCole2* (Figs 7K and 8K) and *TmAtt1b* (Figs 7F and 8F) in response to *E. coli*, *S. aureus*, and *C. albicans* was considerably decreased after *TmDorX2* knockdown.

## Discussion

Insects have evolved a robust tolerance and resistance mechanism against pathogenic infections which enable them to adapt to a wide variety of environmental niches (e.g., endoparasitic lifestyle)^44^. The plasticity of the innate immune defense mechanisms are pivotal towards combating microbial infection^45^. Towards elucidating the biochemical basis of innate immunity, such as the mechanism of pathogen recognition and the ensuing signaling cascades, *D. melanogaster* and *T. castaneum* have been used as reliable insect models. *T. molitor* has recently emerged as an excellent host-pathogen interaction model^24^. Compared to *D. melanogaster*, which is intolerant of high temperatures (25 °C and 37 °C)^46^, *T. molitor* exhibits thermal tolerance, making the species suitable for studying host defense mechanism against biotic and abiotic stressors^47,48^. In addition, laboratory rearing of *T. molitor* is relatively easy^49^ and its transcriptome, which represents the largest genetic sequence dataset for insects, has been already reported^50^, providing possibilities for carrying out molecular studies. A comparison of the Toll signaling pathway between *T. molitor* and *Drosophila* has revealed commonalities and differences in terms of the immune signaling mechanisms. In *Drosophila*, Lys-type PGNs of Gram-positive bacteria, β-1,3-glucan of fungi, and DAP-type PGNs of Gram-negative bacteria, activate the Toll signaling pathway^51,52^. Unlike *Drosophila*, the polymeric DAP-type PGN can also be recognized by the *T. molitor* PGRP-SA/GNBP1, complex leading to the sequential activation of a three-step proteolytic cascade, similar to that activated by Lys-Type PGN (the Imd pathway)^53,54^. Accordingly, in *T. molitor*, the recognition of bacterial and fungal PGN initiates Toll and Imd signaling pathways, which induce the expression of AMP genes^23^. Many intracellular proteins are present in the Toll signaling pathway. In the present study, we have focused on its final component, Dorsal, a transcription factor downstream the Toll pathway that translocates into the nucleus and binds to appropriate motifs in the promoters of specific AMP genes^55^.

Focusing on the *T. molitor* Toll pathway, we identified a Dorsal homolog using the *T. castaneum* Dorsal 2 as a query against the *T. molitor* RNAseq database. Conserved domain analysis of the full-length *TmDorX2* ORF revealed RHD and IPT domains, and an NLS at the C-terminus of the IPT domain. All members of the NF-κB family share the structurally conserved RHD^56^. N-terminal sequences of RHD comprise a recognition loop that is responsible for DNA binding; the C-terminal sequences of RHD are required mainly for dimerization and interaction with inhibitor kappa Kinase (IKK)^57^. Previous studies on NF-κB dimerization found that the IPT domain is crucial for homodimerization, and deleting the IPT domains leads to the degradation of NF-κB precursors^58,59^. *TmDorX2* is destined to translocate into the nucleus, hence, it contains an arginine (R)/lysine (K)-rich NLS (N-P_307_GAL**KRKR**E**K**Y_317_-C)^60^. Sequence alignment of *Tm*DorX2 RHD with that from other insects showed six conserved cysteine residues. The amino acid cysteine is fundamental for forming disulfide bonds, which is responsible for protein folding and stability.

The *TmDorX2* mRNA levels were increased at the late larval and 2-day old pupa stages and reached peak values during adult stages, with the highest expression observed in 3-day-old adults. Prior studies on hormonal regulation of the innate immune response showed that juvenile hormone (JH) and ecdysone, which control development and growth in insects, modulate the expression of immune-induced genes in response to pathogen infection^61^. It is possible that, the fluctuations in the mRNA levels of *TmDorX2* mRNA during different developmental stages are related to these versatile hormones.

*TmDorX2* was expressed primarily in MTs and the fat body and less in hemocytes and the gut. Earlier studies in *Drosophila* have established the fat body (equivalent to the mammalian liver) as the foremost immune responsive organ that synthesizes and secretes AMPs into the hemolymph^62,63^. In addition to the fat body epithelial tissues, including the gut epithelium^64^, reproductive tract, trachea epithelial cells, and MTs (nephridia or kidney analogs)^65,66^, play an important role in immune defense. In the coleopteran model, *Zophobas morio*, the fat body and MTs are versatile tissues that share pivotal functions, such as immunity, detoxification, nitrogen metabolism, and eye pigmentation^67^. MTs are considered independent epithelial immune-responsive sites in insects. Furthermore, earlier studies have shown that genes involved in the Imd pathway are expressed in *Drosophila* MTs, and they lead to the induction of AMPs in response to microbial insults^65,66^. Moreover, Toll-associated transcripts, such as Toll receptors, Spz, Tube, Pelle, and Cactus have been detected in the MTs of *Z. morio* larvae^68^. MTs have also been regarded as immune sites that respond to ecdysone in the presence or absence of pathogenic microbes^69^. Furthermore, the mRNA levels of *TmCactin*, (positive regulator of Cactus degradation and mediator of Dorsal trans-nuclear localization) was found to be higher in MTs of *T. molitor*^35^. The results are indicative of simultaneous expression of *TmCactin* and*TmDorX2* in MTs in response to bacterial challenges

To understand the involvement of *Dorsal* in *T. molitor* innate immunity, we examined the mRNA profiles of *TmDorX2* upon *E. coli*, *S. aureus*, and *C. albicans* challenges. Our observation of increased *TmDorX2* transcript levels in the whole-body, fat body, and hemocytes of the host larvae after infection with *E. coli*, and *S. aureus* is consistent with the findings in the Chinese shrimp, *F. chinensis* showing highly upregulated *FcDorsal* mRNA levels in response to both Gram-positive and Gram-negative bacteria^16^. In our study, the highest expression levels of *TmDorX2* were observed 9 h post-infection in the immune tissues (fat body, hemocytes, and gut). Moreover, the increased *TmDorX2* transcript levels in the hemocytes and gut of the *E. coli-*infected group in comparison with those in S. *aureus* and *C. albicans-*challenged groups, suggest that exposure to *E. coli* (Gram-negative bacteria) accelerates *TmDorX2-*Toll induction. A previous study on the expression of *EsDorsal*, in response to lipopolysaccharides (LPS) from *E. coli*, peptidoglycan (PG) from *S. aureus*, and zymosan (GLU) from *Saccharomyces cerevisae*, showed similar results wherein the *EsDorsal* responses to LPS were higher than those to GLU and PG^19^. As explained before in the case of *Drosophila*, the Toll signaling pathway senses β-1,3-glucan from fungi and lysine-type PGN from Gram-positive bacteria, whereas the Imd pathway is activated in response to DAP-type PGN from Gram-negative bacteria^51^. However, previous studies in *T. molitor* have shown that polymeric DAP-type PGN can be recognized by PGPRP-SA and GNBP1 of the Toll pathway^23^. Moreover, It has been recently reported that activation of the Toll pathway in *T. molitor* during *E. coli* infection can occur through *TmToll-7*^31^. Based on these results, it is not surprising that *TmDorX2* is highly expressed after *E. coli* challenge in *T. molitor* larvae.

To investigate the functional role of *TmDorX2* in mediating humoral immunity through the expression of AMP-encoding genes in *T. molitor*, we first monitored the survival rates of young larvae treated with ds*TmDorX2* upon *E. coli*, *S. aureus*, or *C. albicans* challenge. An RNAi efficiency of 91% was confirmed at day 2 post ds*TmDorX2* injection. Larvae mortality rate was significantly higher after ds*TmDorX2* silencing, reaching 48%, 42%, and 55% after 10 days of exposure to *E. coli, S. aureus* and *C. albicans*, respectively. This suggests that *TmDorX2* has a positive and conserved role in *T. molitor* innate immunity against *S. aureus* and *C. albicans* infection, which is consistent with its role in the Pacific white shrimp, *L. vannamei*^17^. The highest mortality rate was observed in *C. albicans-*infected larvae compared to the *E. coli-* and *S. aureus*-challenged groups. It is possible that such high mortality rates of larvae after *C. albicans* insult are due to the absence of *TmDorX2*, which leads to lower AMP-encoding gene expression

AMPs are evolutionarily conserved effectors with bactericidal and antifungal activities, and are produced when free Dorsal translocates into the nucleus. qRT-PCR data from dsRNA-injected groups followed by pathogen infection were interpreted by describing *TmDorX2* as a positive regulator when the expression of AMP genes was suppressed in ds*TmDorX2*-treated groups compared to ds*EGFP*-treated groups, and as a negative regulator in the opposite scenario. The expression of 11 AMP genes was highly increased in the fat body and gut of ds*EGFP*-injected larvae challenged with *E. coli*, whereas levels of all 11 AMP transcripts were significantly decreased in the ds*TmDorX2*-treated groups after *E. coli* insult. Among all 14 AMPs, the *TmAttacin* family^70^, *TmTene2*^71^, and *TmTene4*^23^ are well-known anti-Gram-negative AMPs. In the *T. molitor* gut, the expression levels of *TmAtt1a*, *−1b* and *−2*, and *TmTene2* and *−4* were dramatically downregulated in the ds*TmDorX2*-treated group. In the fat body and hemocytes of *TmDorX2*-silenced larvae, the levels of all mentioned anti-Gram-negative AMPs except for that of *TmTene2* were downregulated. The expression levels of AMP genes in the fat body and gut of *TmDorX2* knockdown larvae after *E. coli* insult (11 AMPs) were more strongly downregulated than those the expression of AMP genes in hemocytes and MTs (8 AMPs).

*TmDorX2* knockdown decreased the survivability of larvae after *C. albicans* challenge. The increased susceptibility of the larvae can be explained by the fact that 11 AMP genes were markedly decreased in the gut of *TmDorX2* dsRNA-treated *T. molitor*. More specifically, the expression of antifungal AMPs (*TmTene3*, *TmTLP1*, and *TmTLP2*) was strongly upregulated in response to *C. albicans*, and this response was greatly decreased upon *TmDorX2* knockdown. The requirement of *TmTene3* as an antifungal AMP is known^72^, and supports the results of our study. Furthermore, the mRNA levels of *TmTene1* and *TmTene2* observed upon *TmDorX2* knockdown this study agree with previously reported results in the gut^33^. In the fat body and hemocytes, upon *TmDorX2* knockdown, the expression of 10 and 8 AMP genes decreased after *S. aureus* injection, respectively. Taken together, our results suggest that the gut is a crucial immune tissue for mediating an innate immune response to *C. albicans* and *E. coli*, while the fat body is pivotal for conferring defense against *S. aureus* and *E. coli*.

Overall, the expression levels of AMP-encoding genes in *TmDorX2* knockdown larvae significantly decreased after bacterial and fungal challenge compared with those in ds*EGFP*-injected groups. In a previous study, *TmCactin* knockdown led to the downregulation of 7AMP genes namely *TmTene1* and *−4*, *TmDef1* and *−2*, *TmCole1* and *−2*, and *TmAtt1b* post-*E. coli*, *-S. aureus*, and -*C. albicans* challenges^35^. Upon infection with all the above mentioned microbes, ds*TmDorX2*-treated larvae showed significant downregulation of 6, 6, 9, and 2 AMP genes in the fat body, hemocytes, gut and MTs, respectively (Fig. 10). We must add that, similar to the depletion of *TmCactin* and *TmToll-7* genes, *TmDef2* is significantly decreased in the fat body, gut, and MTs tissues of ds*TmDorX2*-treated larvae^31,35^. In addition, we found that the induction of *TmAtt2* was suppressed in the fat body, hemocytes, and gut of *TmDorX2*-silenced larvae; a similar downregulation has been observed in ds*TmToll-7* injected larvae^31^. These findings suggest that *TmDef2* and *TmAtt2* are induced after Toll pathway stimulation mediated by *E. coli*, *S. aureus*, and *C. albicans* exposure.

Interestingly, we found several AMP genes induced in the ds*TmDorX2*-treated group compared to the ds*EGFP*-treated group. These results raise the possibility that *Tm*DorX2 acts as a negative regulator of those AMP genes in different tissues, but also that of cross talk between Toll and another immune signaling pathway, such as the IMD pathway^35^.

Furthermore, ongoing investigations on another transcription factor, *T. molitor Relish* (*TmRelish*), showed that 9 AMP-encoding genes, namely *TmTene2* and *−4*, *TmDef1* and *−2*, *TmCole1* and *−2*, *TmAtt1a* and *−1b*, and *TmCec2*, decreased in the fat body tissue of *TmRelish*-silenced larvae against same pathogens (unpublished data). Consequently, the mRNA levels of *TmAtt1a, TmDef1* and *−2*, and *TmCec2* are regulated by both *TmRelish* (Imd) and *TmDorX2* (Toll) pathway in the fat body.

Finally, the mortality rate of *T. molitor* larvae upon *C. albicans* and *S. aureus* infection was higher that upon exposure to *E. coli* suggesting that *TmDorX2* is required for mounting an innate immune response against *S. aureus* and *C. albicans* in the larval gut followed by a response in the fat body and hemocytes.

## Conclusions

The *Dorsal* homologue identified in *T. molitor* (*TmDorX2*) was highly expressed in the fat body and MTs, and less in the hemocytes and gut. Upon challenge with *E. coli*, *S. aureus*, and *C. albicans*, the *TmDorX2* mRNA levels were highly upregulated in the gut, fat body, and hemocytes of *T. molitor* larvae. According to mortality assay results, survival of *TmDorX2*-silenced larvae was remarkably decreased after *S. aureus* and *C. albicans* infection to a greater extent than after *E. coli* challenge, although the effect was significant in all infected groups. A Loss-of-function study of AMP expression revealed that *TmDorX2* knockdown affects the induction of 11 AMP genes against *E. coli* and *C. albicans* in the larval gut, whereas it downregulates 10 AMP genes in response to *S. aureus* in the fat body. In summary, *TmDorX2* can be considered as a positive regulator against *E. coli*, *S. aureus*, and *C. albicans* in the fat body, hemocytes, gut, and MTs of young *T. molitor* larvae. As inferred, injection of the microorganisms upregulated 6, 6, 9, and 2 AMP genes in fat body, hemocytes, gut and MTs of ds*TmDorX2* larvae, respectively. *TmDorX2* knockdown followed by microbial challenge resulted in high mortality of *T. molitor* larvae due to downregulation of AMPs, suggesting that *TmDorX2* plays a key role against bacterial and fungal infections in immune tissues such as the fat body and gut.

## Supplementary information

Supplementary figures 1 ~ 3
