# Supplementary material for: TmDorX2 positively regulates antimicrobial peptides in Tenebrio molitor gut, fat body, and hemocytes in response to bacterial and fungal infection
Source: Sci Rep. 2019 Nov 14;9:16878. doi: 10.1038/s41598-019-53497-4 (PMC6856108; doi:10.1038/s41598-019-53497-4)
Supplement: Supplementary file 1 — Supplementary figures 1 ~ 3 [file 41598_2019_53497_MOESM1_ESM.docx]

Supplementary Material

***TmDorX2* positively regulates anti-microbial peptides in *Tenebrio molitor* gut, fat body, and hemocytes in response to bacterial and fungal infection**

Maryam Keshavarz^1#^, Yong Hun Jo^1#^, Ki Beom Park^1^, Hye Jin Ko^1^, Tariku Tesfaye Edosa^1^, Yong Seok Lee^2^ and Yeon Soo Han^1*^

^1^Department of Applied Biology, Institute of Environmentally-Friendly Agriculture (IEFA), College of Agriculture and Life Sciences, Chonnam National University, Gwangju 61186, Republic of Korea.

^2^ Department of Life Science and Biotechnology, College of Natural Sciences, Soonchunhyang University, Asan, South Korea

^#^ These authors contributed equally to this work

*** Correspondence:**
Yeon Soo Han
[hanys@jnu.ac.kr](mailto:hanys@jnu.ac.kr)

# Supplementary Figure 1


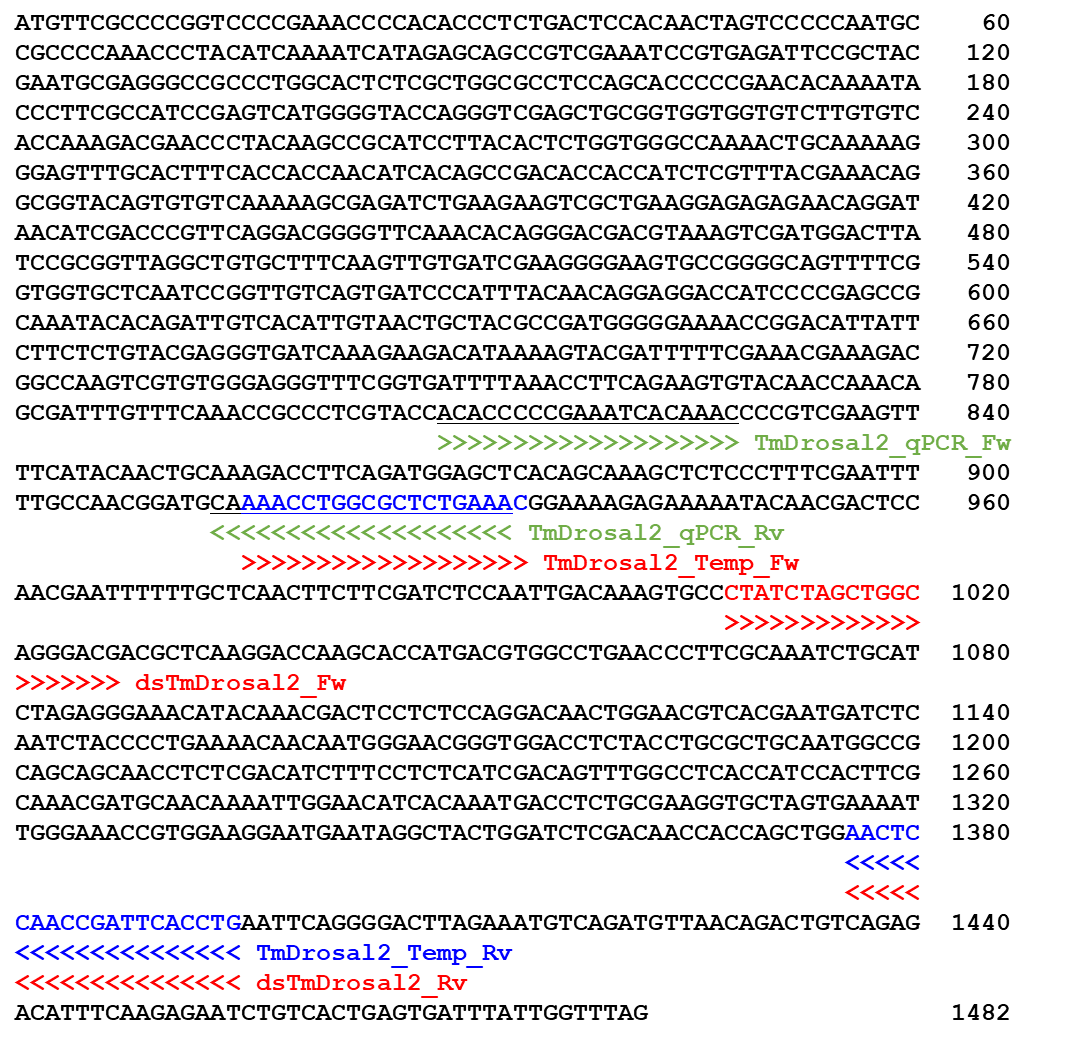


**Supplementary Figure 1.** The full length cDNA sequence of *TmDorX2*. The position of all primers used for real-time analysis and dsRNA synthesis are marked.

# Supplementary Figure 2


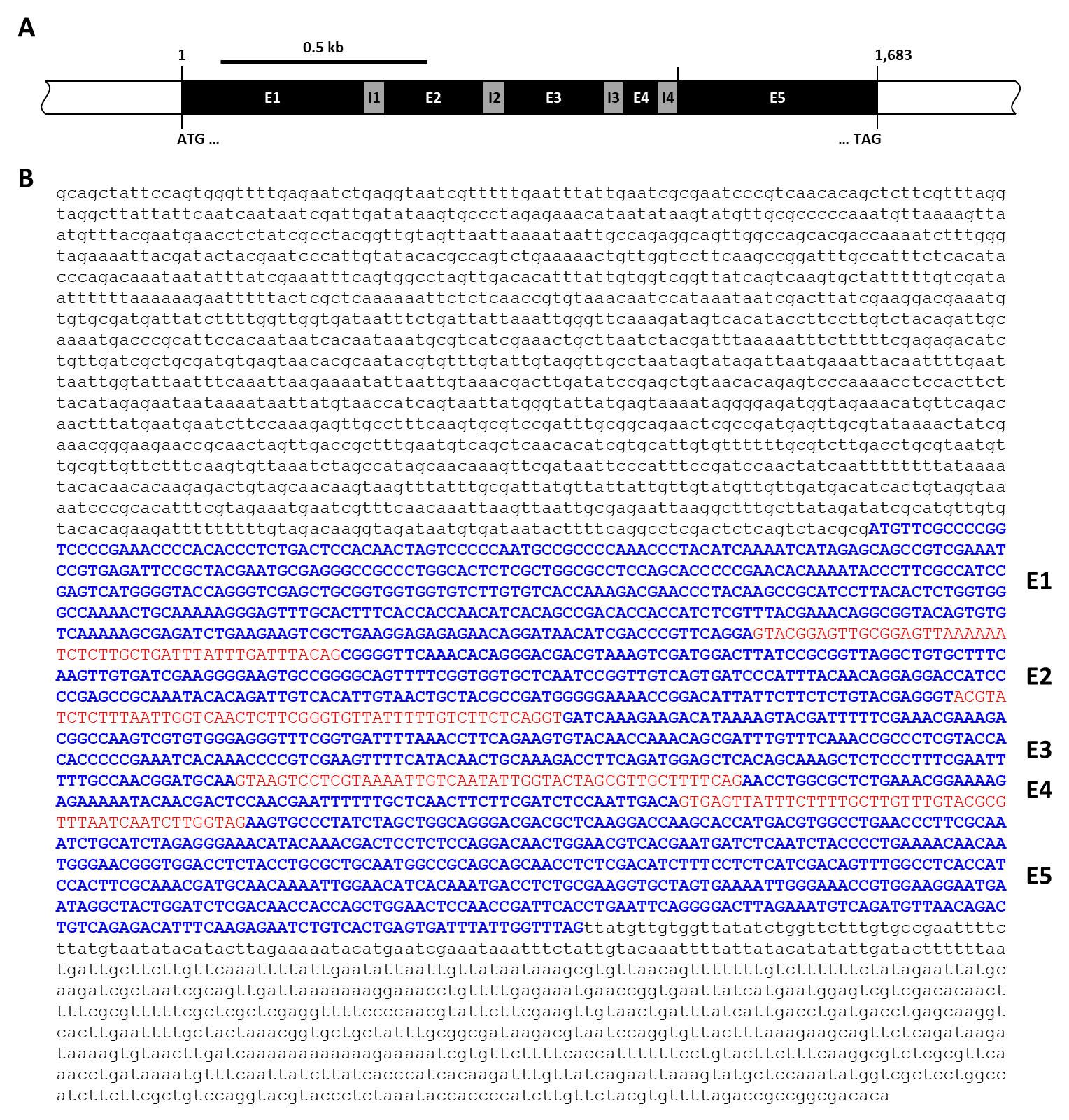


**Supplementary Figure 2.** Schematic illustration of the genomic organization and sequence of *Tenebrio molitor* Dorsal protein isoform X2 (*TmDorX2*). *TmDorX2* representation gene structure shows the promotor, exon (E1-E5) and intron (I1-I4) regions. The translation start (**ATG**) and stop codon (**TAG**) are labeled (**A**). Nucleotide sequence of *TmDorX2* exhibits 5 exons in blue color and 4 introns in red color (**B**).

# Supplementary Figure 3


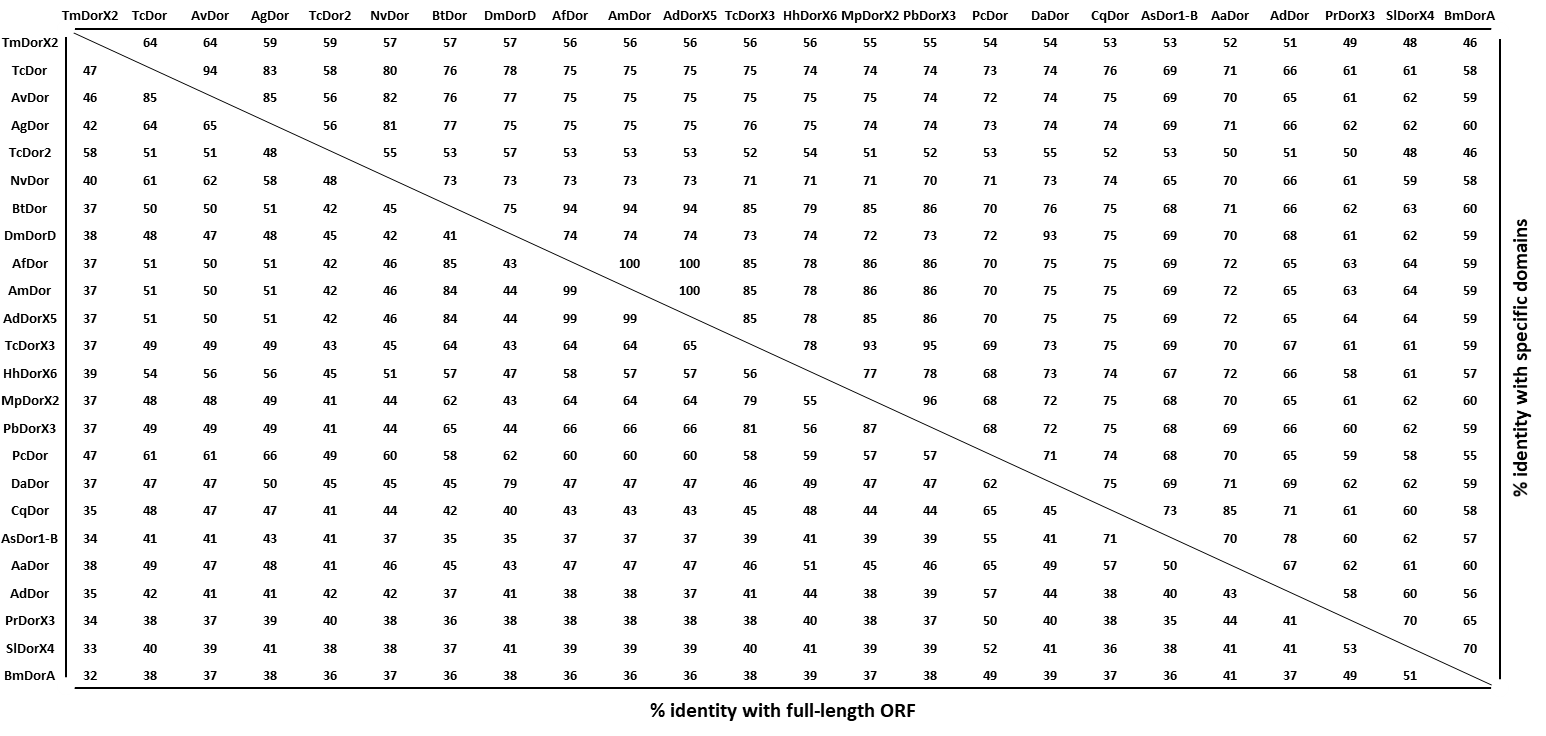


**Supplementary Figure 3.** Percentage identity with full-length ORF and specific conserved domains of *TmDorX2* and other insects.
